# Effects of intensive blood pressure control on mortality and cardiorenal function in chronic kidney disease patients

**DOI:** 10.1080/0886022X.2021.1920427

**Published:** 2021-05-10

**Authors:** Yong Zhang, Jing-Jing Li, An-Jun Wang, Bo Wang, Shou-Liang Hu, Heng Zhang, Tian Li, Yan-Hong Tuo

**Affiliations:** a Department of Nephrology, Jianli People's Hospital, Jingzhou, China; b Department of Ultrasonic Imaging, The Central Hospital of Wuhan, Tongji Medical College, Huazhong University of Science and Technology, Wuhan, China; c Department of Ultrasound, The First Medical Center, Chinese People’s Liberation Army General Hospital, Beijing, China; d Department of Nephrology, The First Affiliated Hospital of Yangtze University, Jingzhou, China; e Department of Histology and Embryology, Xiang Ya School of Medicine, Central South University, Changsha, China; f School of Basic Medicine, Fourth Military Medical University, Xi’an, China; g Department of Nephrology, The Central Hospital of Wuhan, Tongji Medical College, Huazhong University of Science and Technology, Wuhan, China

**Keywords:** Chronic kidney disease, intensive BP control, renal outcomes, cardiovascular outcomes, meta-analysis

## Abstract

**Background:**

Blood pressure (BP) variability is highly correlated with cardiovascular and kidney outcomes in patients with chronic kidney disease (CKD). However, appropriate BP targets in patients with CKD remain uncertain.

**Methods:**

We searched PubMed, Embase, and the Cochrane Library for randomized controlled trials (RCTs) of CKD patients who underwent intensive BP management. Kappa score was used to assess inter-rater agreement. A good agreement between the authors was observed to inter-rater reliability of RCTs selection (kappa = 0.77; *P* = 0.005).

**Results:**

Ten relevant studies involving 20 059 patients were included in the meta-analysis. Overall, intensive BP management may reduce the incidence of cardiovascular disease mortality (RR: 0.69, 95% CI: 0.53 to 0.90, *P*: 0.01), all-cause mortality (RR: 0.77, 95% CI: 0.67 to 0.88, *P* < 0.01) and composite cardiovascular events (RR: 0.84 95% CI: 0.75 to 0.95, *P* < 0.01) in patients with CKD. However, reducing BP has no significant effect on the incidence of doubling of serum creatinine level or 50% reduction in GFR (RR: 1.26, 95% CI: 0.66 to 2.40, *P* = 0.48), composite renal events (RR 1.07, 95% CI: 0.81 to 1.41, *P* = 0.64) or SAEs (RR: 0.97, 95% CI: 0.90 to 1.05, *P* = 0.48).

**Conclusion:**

In patients with CKD, enhanced BP management is associated with reduced all-cause mortality, cardiovascular mortality, and incidence of composite cardiovascular events.

## 1. Introduction

Hypertension is a common clinical manifestation of chronic kidney disease (CKD) and one of the main causes of end-stage renal disease (ESRD) [1,2]. Several studies have shown that intensive blood pressure (BP) control reduces mortality and cardiovascular risk [3,4]. However, the effect of lower BP targets on CKD remains unclear [5,6] because hypotension may lead to a decreased risk of acute kidney injury (AKI) and a rapid decline in the estimated glomerular filtration rate (eGFR) [7,8].

Previous clinical practice guidelines have indicated BP targets for patients with CKD. The 2012 Kidney Disease: Improving Global Outcomes (KDIGO) BP clinical practice guideline suggests a target BP <130/80 mmHg for CKD patients with severe proteinuria and a target BP <140/90 mmHg for CKD patients with proteinuria <30 mg/day [9]. The 2013 European Society of Cardiology Task Force16 [10] and the Eighth Joint National Committee [3] suggest that BPof CKD patients should be less than 140/90 mmHg, whereas it made no distinctions regarding the albuminuria levels [11–13]. However, subsequent studies in people with CKD have yielded inconsistent conclusions [8,14,15]. As a result, clinicians are still unsure of the optimal BP level for patients with CKD.

The worldwide incidence of CKD is 8–16%, and CKD increases the risk of cardiovascular disease and death [16,17]. Many previous studies have shown that maintaining the BP at an acceptable level reduces the risk of kidney damage and cardiovascular death [18,19]. Antihypertensive therapy in patients with CKD is designed to reduce the risk of BP-related cardiovascular disease and delay the progression of renal disease. Previous guidelines recommended that BP should be less than 140/80 mmHg in patients with CKD [13,20]. However, due to a lack of reliable patient data in clinical research, the new guidelines for CKD patients who also have high BP give BP targets that are more conservative than in the past. In 2012, KDIGO advised that CKD patients without albuminuria should maintain their BP at 140/90 mmHg or less, and those with proteinuria or albuminuria should maintain their BP at 130/80 mmHg or less [21]. Elevated systolic BP is the most common manifestation of hypertension in CKD patients and is a unique risk factor for the occurrence and progression of CKD [22]. However, there is still no consensus on the long-term renal benefits of controlling the systolic BP at ≤140 mmHg or lower in patients with CKD.

Here, we conducted a pooling analysis of randomized controlled studies (RCTs) to evaluate effects of intensive BP control on mortality, renal function and cardiovascular events in CKD patients.

## 2. Methods

### 2.1. Protocol

This article is conducted in accordance with the Preferred Reporting Items for Systematic Reviews and Meta-Analyses (PRISMA) guideline [23] and registered in IMPLASY (DOI: 10.37766/inplasy2020.3.0001).

### 2.2. Search strategy

Two reviewers independently searched the Cochrane Collaboration, PROSPERO, and INPLASY database to avoid any duplicates in published meta-analyses. They independently performed a comprehensive literature search in PubMed, EMBASE and the Cochrane Library *via* medical subject heading (MeSH), Emtree and text word with no language limitations from inception to Feb, 2021. The following keywords were used: ‘CKD’, ‘anti-hypertensive agents’, ‘“intensive BP treatment’ and ‘strict blood pressure control’. Reference lists from the included studies were also searched for potentially eligible articles. Search strategy is shown in Supplementary Table S1.

### 2.3. Eligibility criteria

Two authors reviewers independently carried out the primary review to search for all potentially relevant studies. Any disagreements were solved by discussion or consultation with a third author (Tuo) (Supplementary Table S2).

The following criteria were applied:

1. Only randomized controlled trials (RCTs) were included in the study.
2. Participants were patients with CKD or included in a subgroup of CKD.
3. Participants were over 18 years of age.
4. One of the following outcomes must have been included: cardiovascular disease death, all-cause mortality, composite cardiovascular events, composite renal outcome, serum creatinine level doubling, 50% reduction in GFR, or serious adverse events (SAEs). The main characteristics of the included RCTs are summarized in Table 1.
5. Different antihypertensive targets had to be included in the study. The experimental group was the intensive BP control group or the antihypertensive drug treatment group, and the control group was the standard BP group or the placebo group.

**Table 1.** Main characteristics of the included studies.

Study	Year	Region	Design	Trial method(s)	Sample Size (n)		Age		Target BP(mmHg)		Follow-up (years)
					Intensive	Standard	Intensive	Standard	Intensive	Standard	
Aggarwal	2019	USA	RCT	MDRD, AASK, ACCORD and SPRINT	2509	2474	64 ± 13.5	64 ± 13.6	SBP < 130	SBP < 140	3.5
Cheung	2017	USA	RCT	SPRINT	1330	1316	72.0 ± 9.0	71.9 ± 9.5	SBP < 120	SBP < 140	3.3
E-KU	2014	USA	RCT	MDRD	171	153	51.5 ± 12.6	52.0 ± 12.2	MBP < 92	MBP < 107	6.0
Hayashi	2010	Japan	RCT	JATOS	1230	1269	73.6 ± 5.3	72.9 ± 4.9	SBP < 140	SBP < 160	2.0
Lambers	2010	Australia	RCT	ADVANCE	1020	1013	65.3 ± 6.2	65.0 ± 6.4	NA	NA	4.3
Malhotra	2019	USA	RCT	SPRINT	519	419	72.0 ± 9.0	72.0 ± 9.0	SBP < 120	SBP < 140	4.0
Mezue	2018	USA	RCT	SPRINT	1215	1273	72.0 ± 9.4	72.0 ± 8.9	SBP < 120	SBP < 140	2.2
Ogihara	2010	Japan	RCT	VALISH	477	467	76.1 ± 4.1	76.1 ± 4.1	SBP < 140	SBP: 140-150	2.9
Schrier	2014	USA	RCT	HALT-PKD	274	284	36.9 ± 8.2	36.3 ± 8.4	95/60 to 110/75	120/70 to 130/80	8.0
Wright	2015	USA	RCT	SPRINT	1330	1316	67.9 ± 9.4	67.9 ± 9.5	SBP < 120	SBP < 140	6.0

AASK: African American Study of Kidney Disease and Hypertension. ACCORD: Action to Control Cardiovascular Risk in Diabetes. ADVANCE: Action in Diabetes and Vascular Disease. HALT-PKD: The Polycystic Kidney Disease Treatment Network. JATOS: Japanese Trial to Assess Optimal Systolic Blood Pressure in Elderly Hypertensive patients. MDRD: Modification of Diet in Renal Disease. SPRINT: Systolic Blood Pressure Intervention Trial. VALISH: Valsartan in Elderly Isolated Systolic Hypertension Study.

### 2.4. Data extraction

Two reviewers independently extracted data from enrolled studies: first author, publication year, author's nationality, type of trial design, trial method(s), sample size, age, target BP and follow-up time. Primary results are defined as all-cause mortality, renal outcomes and cardiovascular outcomes. Secondary result included serious adverse events (SAEs).

### 2.5. Risk of bias assessment

Two authors (Li and Zhang) independently assessed the quality of all RCTs according to the twelve criteria recommended by the Cochrane Back Review Group [24] (Supplementary Figure S1). Any disagreements between the authors (Li and Zhang) were resolved through discussion with a third author (Tuo). We used weighted kappa scores to assess inter-rater agreements. If a kappa score below 0.2 was considered a ‘none’ agreement, 0.21–0.39 as ‘minimal’ agreement, 0.40–0.59 as ‘weak’ agreement, 0.60–0.79 as ‘good’ agreement, 0.80–0.90 as ‘strong’ agreement, above 0.90 as ‘almost perfect’ agreement [25].

**Figure 1.**
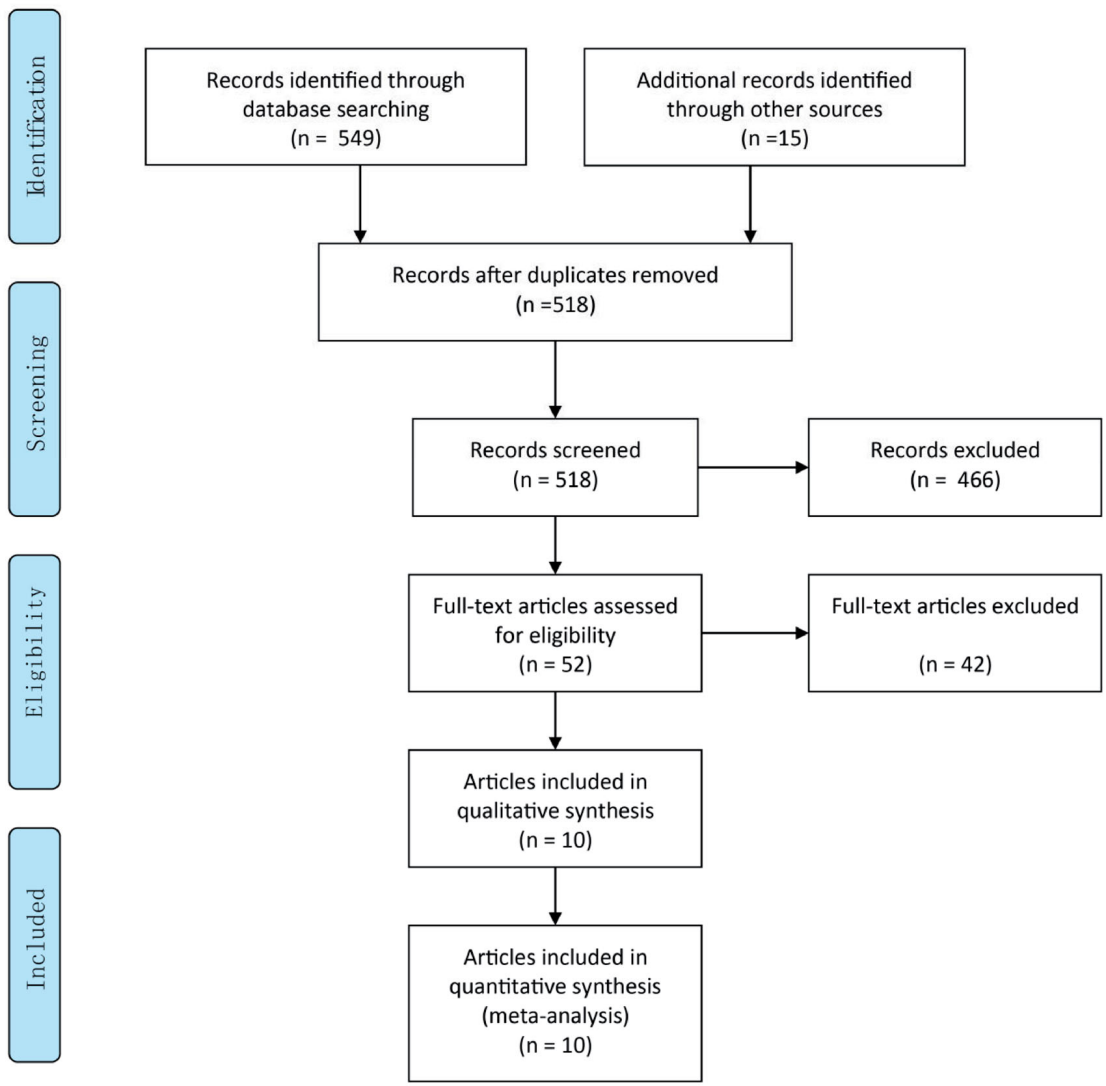
PRISMA 2009 flow diagram.

### 2.6. Statistical analysis

STATA 16.0 (Stata Corp LP, College Station, TX) was used to perform statistical analyses in a fixed-effects model. Relative ratios (RRs) with 95% confidence intervals (CIs) were used as the effect size measures of dichotomous data. Heterogeneity was analyzed by *I*^2^. Heterogeneity was categorized as follows: low, *Ι*^2^ = 0–25%; medium, Ι^2^ = 25–50%; high, *Ι*^2^ = 50–75%; and powerful, Ι^2^ = 75–100%; an *Ι*^2^ less than 50% was considered to represent tolerable heterogeneity [26]. If there was significant heterogeneity, a sensitivity analysis was conducted to evaluate the consistency and quality of the results.

## 3. Results

### 3.1. Study selection

We identified and screened 518 potentially relevant articles in the initial retrieval, after excluding duplicate records (*n* = 46). Fifty-two articles were retained after title/abstract curation (excluding 466 records). Thereafter, we read the full text and finally, 10 RCTs [8,27–35] involving a total of 20 059 patients were included in the meta-analysis, as listed in Table 1. Our search strategy is described in Figure 1. The kappa’s score for inter-rater agreement of study selection was 0.77 (95% CI 0.23 − 1.31), which demonstrated ‘good’ inter-rater agreement. Inter-rater reliability for risk of bias assessment were provided in Table 2.

**Table 2.** Inter-rater agreement for study selection and risk of bias.

Risk of bias	95% confidence interval	Kappa	*P*	Agreement (%)
Study selection	0.23–1.31	0.77	0.005	86
Random sequence generation (selection bias)	0.40–1.89	0.84	0.0001	90
Allocation concealment (selection bias)	0.28–1.12	0.70	0.001	80
Blinding of participants and personnel (performance bias)	0.21–1.23	0.67	0.004	90
Blinding of outcome assessment (detection bias)	0.40–1.89	0.84	0.0001	90
Incomplete outcome data (attrition bias)	0.10–1.17	0.64	0.02	90
Selective reporting (reporting bias)	0.40–1.89	0.84	0.0001	90

### 3.2. All-cause mortality

**Figure 2.**
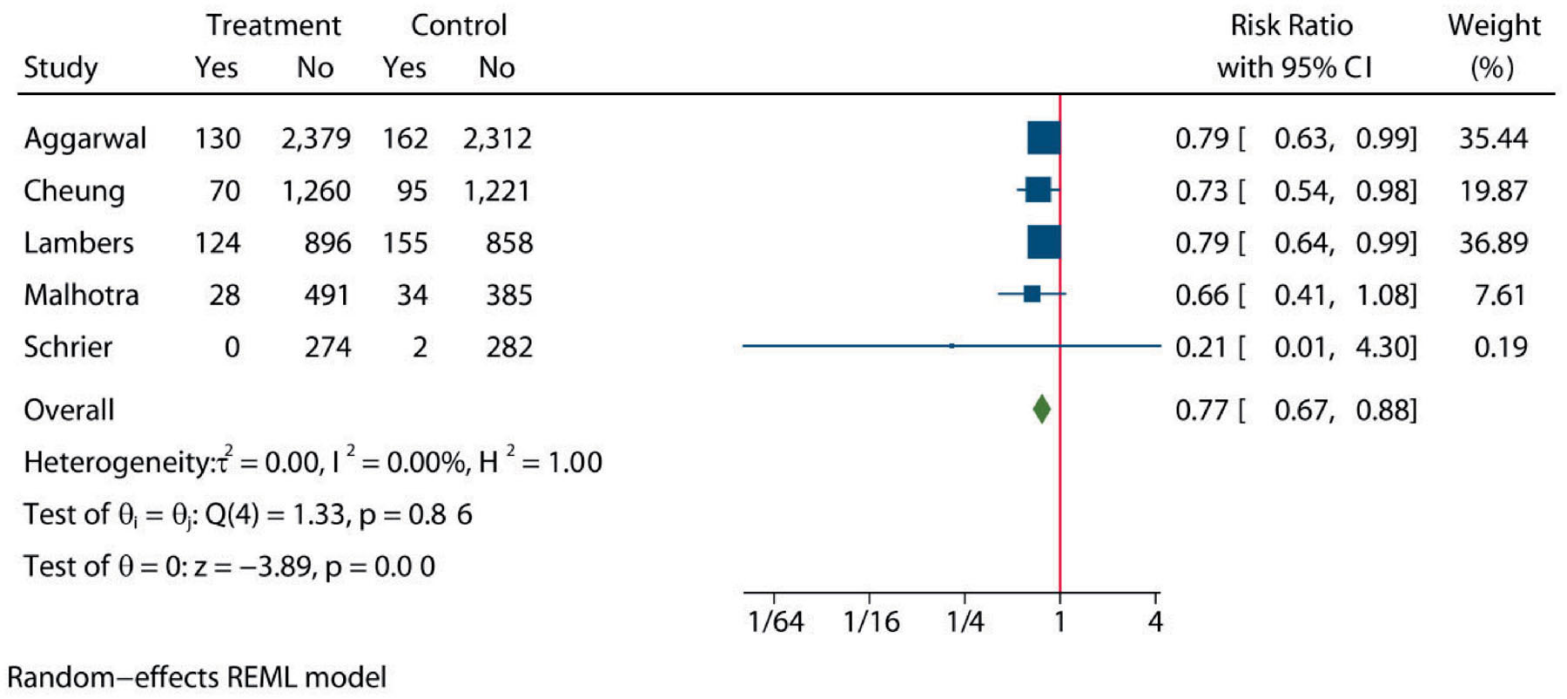
Forest plot of all-cause mortality.

**Figure 3.**
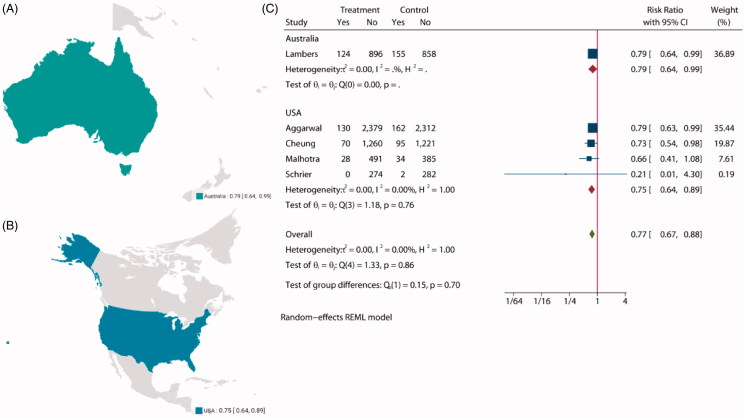
The choropleth map of all-cause mortality.

Pooled analysis of the incidence of all-cause mortality was reported in five studies [8,27,28,30,32] involving 11 158 patients, with 5652 assigned to intensive BP lowering groups and 5506 assigned to standard groups. Compared with patients in the standard groups, patients in the intensive BP lowering groups showed a statistically significant decrease in the incidence of all-cause mortality (RR: 0.77, 95% CI: 0.67 to 0.88, *p* < 0.01, Figure 2), and funnel plot is presented in Supplementary Figure S2. The choropleth map reveals that regional difference of all-cause mortality in Australia and USA (Figure 3). Overall, intensive BP management may reduce the incidence of all-cause mortality in USA, but the difference was not significant in Australia, which may be due to the small sample size included in the Australia study. We also conducted a subgroup analysis with follow-up (Supplementary Figure S3), sample size (Supplementary Figure S4), target SBP (Supplementary Figure S5) in treatment group and control group. Overall, no evidence suggests that the observed effects of more intensive BP-lowering regimens on all-cause mortality differed across trial subgroups defined according to a broad range of study characteristics.

The meta-regression by Bubble plot reveals that publication year (*p* < 0.01, Supplementary Figure S6) and sample size (*p* = 0.03, Supplementary Figure S7) may cause potential sources of heterogeneity. Sensitivity analysis (Supplementary Figure S8) was performed to evaluate the stability of our results. The analysis results suggested that no individual studies significantly affected the pooled OR, indicating that the results were statistically robust.

### 3.3. Cardiovascular outcomes

#### 3.3.1. Cardiovascular disease death

**Figure 4.**
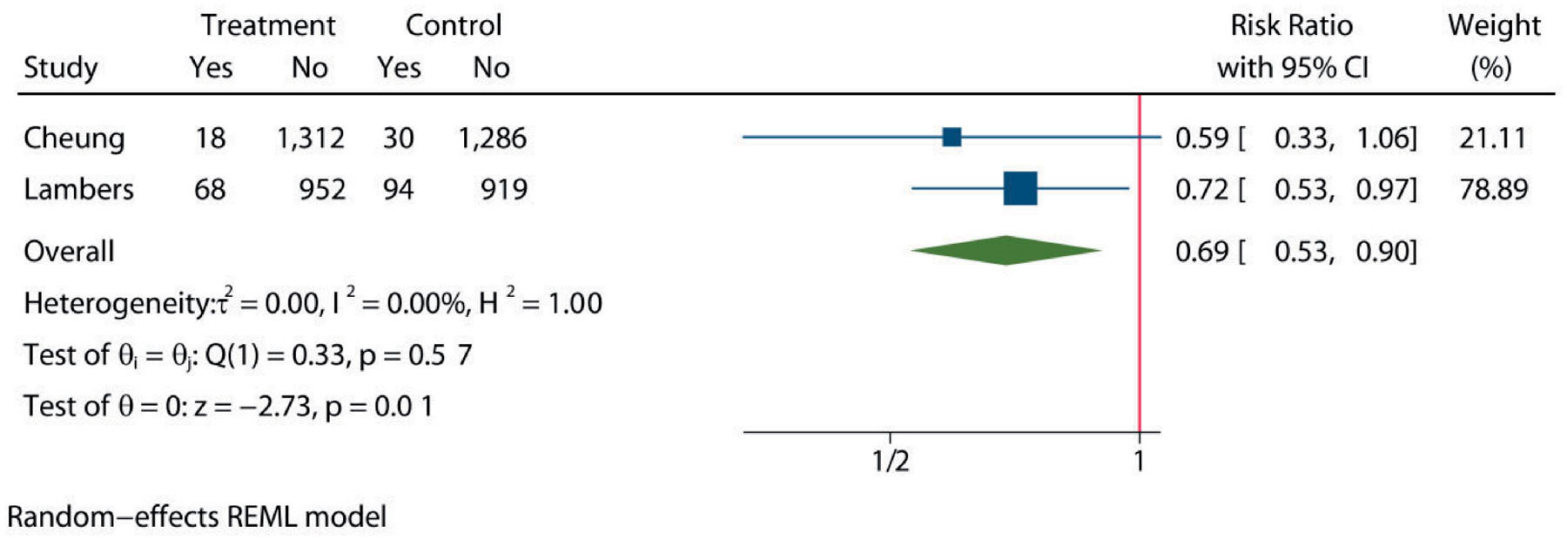
Forest plot of cardiovascular disease death.

Two studies [28,30] assessed cardiovascular disease death in a total of 4679 patients, 2350 of whom were assigned to an intensive BP lowering group and 2329 to a standard group. The statistical analysis showed a lower incidence of cardiovascular disease death in the intensive BP lowering group than in the standard group (RR: 0.69, 95% CI: 0.53 to 0.90, *P* = 0.01, Figure 4), and funnel plot is shown in Supplementary Figure S9.

#### 3.3.2. Composite cardiovascular events:

**Figure 5.**
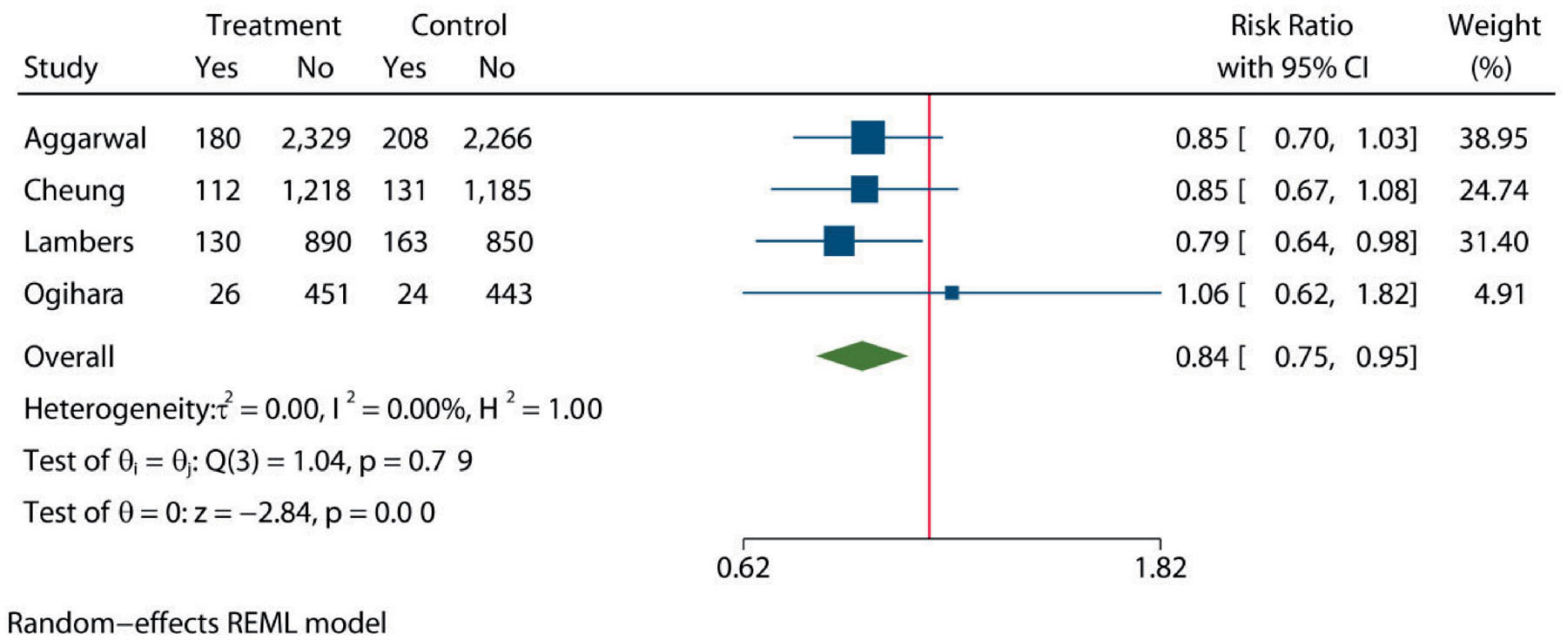
Forest plot of composite cardiovascular events.

Four studies [27,28,30,34] reported the incidence of cardiovascular events in a total of 9347 patients, 4694 of whom were assigned to an intensive BP lowering group and 4653 to a standard group. Composite cardiovascular events including heart failure, stroke and vascular disease. The statistical analysis showed a lower incidence of composite cardiovascular events in the intensive BP lowering group than in the standard group (RR: 0.84, 95% CI: 0.75 to 0.95, *p* < 0.01, Figure 5), and funnel plot is shown in Supplementary Figure S10.

### 3.4. Renal outcomes

#### 3.4.1. Doubling of serum creatinine level or 50% reduction in GFR

**Figure 6.**
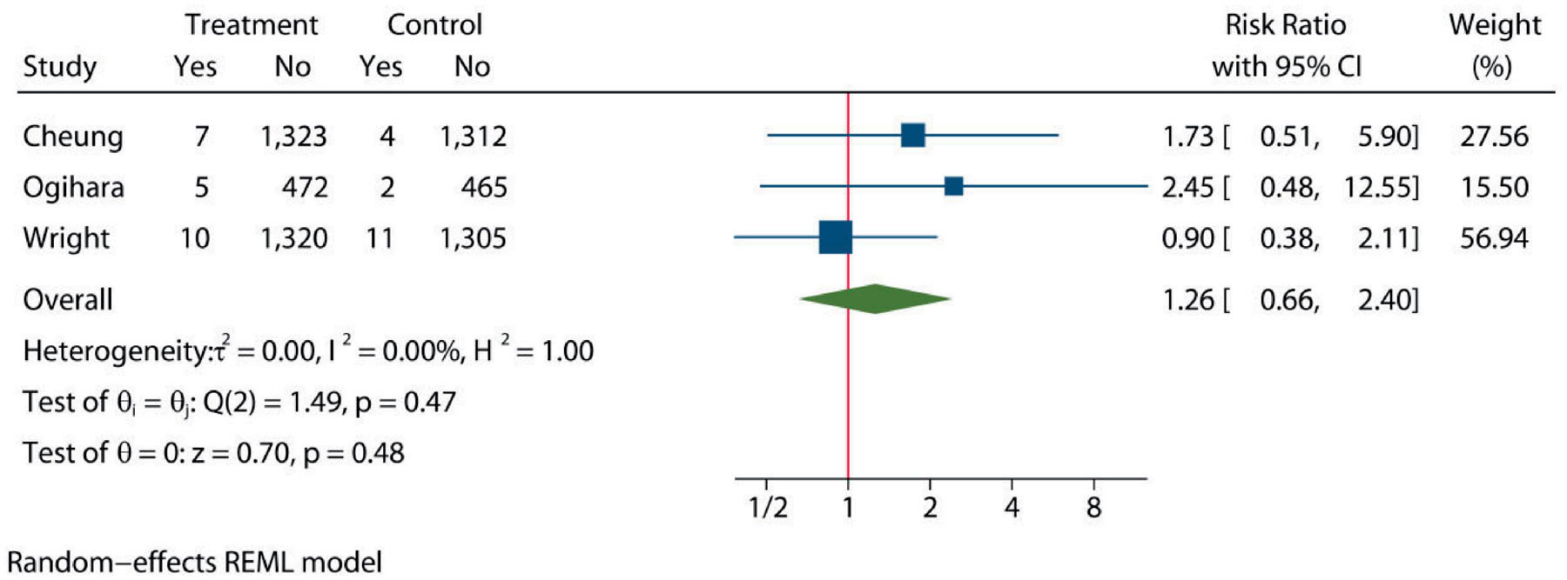
Forest plot of doubling of serum creatinine level or 50% reduction in GFR.

Three studies [28,34,35] assessed the incidence of doubling of serum creatinine level or a 50% reduction in the GFR in a total of 6236 patients, 3137 of whom were assigned to an intensive BP lowering group and 3099 to a standard group. The statistical analysis showed no significant difference between the two groups (RR: 1.26, 95% CI: 0.66 to 2.40, *P* = 0.48, Figure 6), and funnel plot is shown in Supplementary Figure S11.

#### 3.4.2. Composite renal outcome

**Figure 7.**
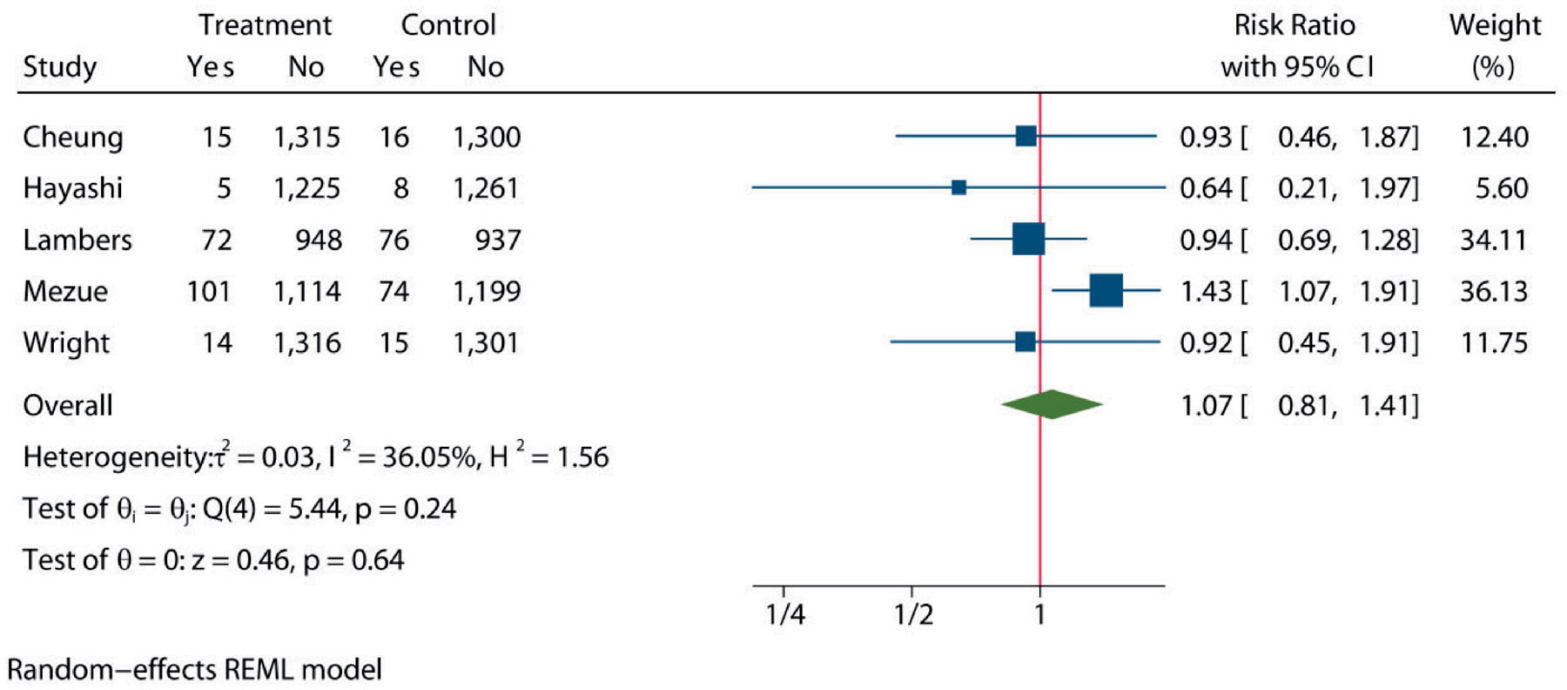
Forest plot of composite renal outcome.

**Figure 8.**
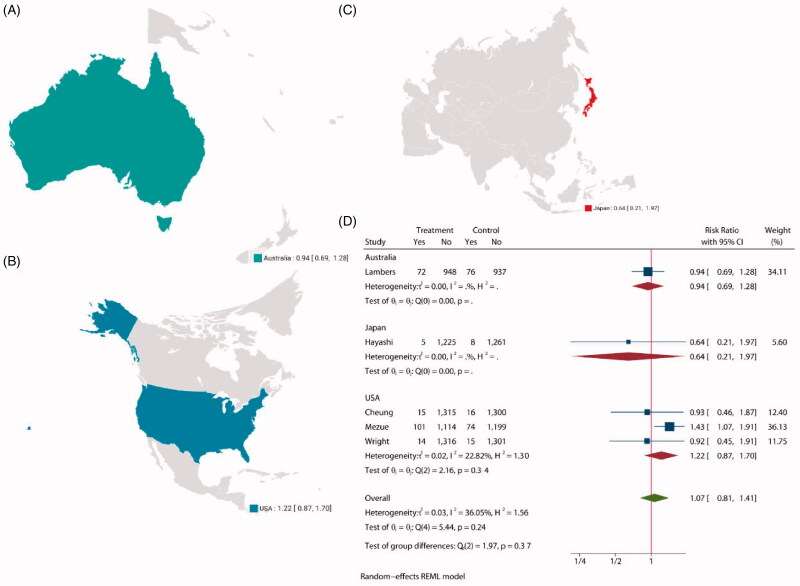
The choropleth map of composite renal outcome.

Five studies [28–30,33,35] involving 12 312 participants recorded a total of 396 kidney failure events during treatment, with 6125 assigned to intensive BP lowering groups and 6187 assigned to standard groups. Composite renal outcome including new or worsening nephropathy (development of macroalbuminuria), need for renal replacement therapy, or death due to renal disease. The statistical analysis showed no significant difference between the two groups (RR: 1.07, 95% CI: 0.81 to 1.41, *P* = 0.64, Figure 7) in patients with CKD, and funnel plot is shown in Supplementarry Figure S12. The choropleth map reveals that no regional difference of composite renal outcome in Australia Japan and USA (Figure 8). We also conducted a subgroup analysis with follow-up (Supplementary Figure S13) and target SBP (Supplementary Figure S14) in treatment group and control group. Overall, no evidence suggests that the observed effects of more intensive BP-lowering regimens on composite renal outcome differed across trial subgroups defined according to a broad range of study characteristics.

The meta-regression by Bubble plot indicated that no significant heterogeneity of the publication year (*p* = 0.10, Supplementary Figure S15), and sample size (*p* < 0.01, Supplementary Figure S16) was a potential major source of heterogeneity. Sensitivity analysis (Supplementary Figure S17) was performed to evaluate the stability of our results. The analysis results suggested that no individual studies significantly affected the pooled OR, indicating that the results were statistically robust.

### 3.5. SAEs

**Figure 9.**
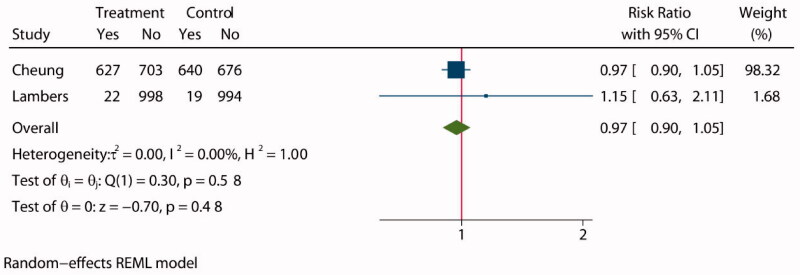
Forest plot of serious adverse events.

SAEs included events that were fatal required hospitalization, such as hypotension, syncope, bradycardia, injurious falls, hyponatremia, hypernatremia, or orthostatic hypotension. Only two studies [28,30] reported the incidence of SAEs during treatment in a total of 4679 patients, with 2350 assigned to intensive BP lowering groups and 2329 assigned to standard groups. The statistical analysis showed no significant difference between the two groups (RR: 0.97, 95% CI: 0.90 to 1.05, *P* = 0.48, Figure 9), and funnel plot is presented in Supplementary Figure S18.

## 4. Discussion

The current systematic review and meta-analysis of 10 RCTs demonstrated that there was statistically significant decrease incidence of all-cause mortality, cardiovascular mortality and composite cardiovascular events in patients with CKD. These beneficial effects were consistent with major subgroups analysis. Moreover, our results indicate that intensive BP control has no significant effect on the incidence of doubling of serum creatinine level or 50% reduction in GFR, composite renal events and SAEs.

A previous meta-analysis had similar results to our study. Tsai et al. found that intensive BP control could reduce the mortality of non-diabetic patients with CKD. However, their study also found that intensive BP control did not show a significant difference in the change in doubling of serum creatinine level or 50% reduction in GFR, ESRD, composite renal outcome or all-cause mortality, this is different from our conclusion. The results of the meta-analyses were yielded from some data for more than 20 years ago with high risk of bias. Furthermore, the excessively limited target population range and relatively few RCTs may limited their conclusions. To the best of our knowledge, this study is the first one involving representative populations with CKD to meta-analyze the relationship between intensive BP on renal function and cardiovascular events from latest high quality RCTs, rather than previous separated or partial one.

Several previous observational studies have found the association between intensive BP control with cardiovascular and renal events. However, several non-randomized studies in recent years have suggested a J-curve association between BP and outcome. This finding has led to concern that intensive BP control may increase the risk of SAEs. The results of our meta analysis support the idea that moderate BP control does not increase the risk of SAEs. A recent research by Juraschek et al. found that intensive BP-lowering treatment decreases risk for orthostatic hypotension, before or in the setting of more intensive BP treatment, should not be viewed as a reason to avoid or de-escalate treatment, the study also shows that intensive antihypertensive therapy is safe from another aspect.

This study assessed the impact of intensive antihypertensive therapy in patients with CKD, including the incidence of renal events, cardiovascular events, and adverse events. The results showed that intensive hypertension management can reduce the cardiovascular disease mortality, all-cause mortality, and incidence of composite cardiovascular events in CKD patients. However, there were no significant differences in the level of serum creatinine or in the incidence of renal events. There was also no clear evidence that intensive BP control can increase the risk of adverse events.

## 5. Strengths and limitations

Firstly, this article was performed by a Cochrane Member and supervised by strict quality control evaluated by Cohen's kappa coefficient. The primary advantage of our meta-analysis is that it included a large number of high-quality RCTs. For patients with CKD, the decline of renal function and cardiac function is generally relatively slow. Therefore, long-term follow-up is needed to obtain sufficient and comprehensive data. All of the included trials had long-term follow-up data, including a large number of end-stage renal and cardiovascular events. Because the number and quality of the included studies was high and the heterogeneity among the studies was low, the meta-analysis results are relatively reliable.

This study also has some limitations. First, there were differences in the characteristics of the included patients and in the study designs. Some patients with diabetes were included in the RCTs, and thus there was not a homogenous CKD patient population, which may influence the results. Second, some included studies contained only subsets of data. Furthermore, the etiology and proteinuria status of patients with CKD was not clear, and there were also differences among the subjects from different trials, which may have impacted the results. Third, the BP targets in each study were not exactly the same, and different drug treatments in the studies may have led to biased results. Hence, it is not possible to provide a clear target for BP reduction. Fourth, only published studies were included, and although there was no significant publication bias, such bias may nevertheless exist. Therefore, the results require further verification, and additional studies with a strict design and uniform standards are needed to prove a positive link between enhanced hypertension control and renal and cardiovascular outcomes.

## 6. Conclusion

In summary, enhanced BP management is associated with reduced all-cause mortality, cardiovascular mortality, and incidence of composite cardiovascular events in patients with CKD. However, it has no significant effect on the incidence of serum creatinine level doubling, a 50% reduction in GFR, or composite renal events. We hope that our data will provide some useful information on BP management in patients with CKD.

## Supplementary Material

Supplemental Material

Supplemental Material

Supplemental Material

Supplemental Material

Supplemental Material

Supplemental Material

Supplemental Material

Supplemental Material

Supplemental Material

Supplemental Material

Supplemental Material

Supplemental Material

Supplemental Material

Supplemental Material

Supplemental Material

Supplemental Material

Supplemental Material

Supplemental Material

Supplemental Material

Supplemental Material
