# Supplementary material for: Effects of intensive blood pressure control on mortality and cardiorenal function in chronic kidney disease patients
Source: Ren Fail. 2021 May 10;43(1):811–20. doi: 10.1080/0886022X.2021.1920427 (PMC8118417; doi:10.1080/0886022X.2021.1920427)
Supplement: Supplemental Material [file IRNF_A_1920427_SM3673.pdf]

Table S1. Search strategy

Database	No	PICOS	Strategy
PubMed	#1	P	Renal Insufficiency, Chronic [MeSH] OR Chronic Renal Insufficiency OR Chronic Kidney Insufficiency OR Chronic Kidney Disease OR Chronic Kidney Diseases OR Chronic Renal Disease OR Chronic Renal Diseases
	#2	I	Blood Pressure Monitors [MeSH] OR Monitors, Blood Pressure OR Monitors, Blood Pressure OR Blood Pressure Monitor OR Blood Pressure Determination OR Blood Pressure Control OR Control Blood Pressure
	#3	C	NA
	#4	O	Mortality [MeSH] OR Cardiovascular Diseases [MeSH] OR Renal Insufficiency [MeSH] OR Heart Arrest OR Heart Failure OR Cardiac Failure OR Heart Decompensation OR Heart Failure, Right-Sided OR Right Sided Heart Failure OR coronary heart diseases OR Myocardial Failure OR Left Sided Heart Failure OR All-cause mortality OR Serum Creatinine OR GFR OR eGFR OR Chronic Kidney Diseases OR glomerular filtration rate OR Composite renal outcome
	#5	S	(randomized controlled trial[Publication Type] OR randomized[TIAB] OR randomised[TIAB] OR placebo[TIAB])) NOT (Review[Publication Type]) NOT (meta-analysis[Publication Type]) NOT (Comment[Publication Type]) NOT (Letter[Publication Type])
	#6 = #1AND#2 AND#4 AND #5	NA	(((((Renal Insufficiency, Chronic [MeSH] OR Chronic Renal Insufficiency OR Chronic Kidney Insufficiency OR Chronic Kidney Disease OR Chronic Kidney Diseases OR Chronic Renal Disease OR Chronic Renal Diseases)) AND ((Blood Pressure Monitors [MeSH] OR Monitors, Blood Pressure OR Monitors, Blood Pressure OR Blood Pressure Monitor OR Blood Pressure Determination OR Blood Pressure Control OR Control Blood Pressure)) AND ((Mortality [MeSH] OR Cardiovascular Diseases [MeSH] OR Renal Insufficiency [MeSH] OR Heart Arrest OR Heart Failure OR Cardiac Failure OR Heart Decompensation OR Heart Failure, Right-Sided OR Right Sided Heart Failure OR coronary heart diseases OR Myocardial Failure OR Left Sided Heart Failure OR All-cause mortality OR Serum Creatinine OR GFR OR eGFR OR Chronic Kidney Diseases OR glomerular filtration rate OR Composite renal outcome)) ((randomized controlled trial[Publication Type] OR randomized[TIAB] OR randomised[TIAB] OR placebo[TIAB])) NOT (Review[Publication Type]) NOT (meta-analysis[Publication Type]) NOT (Comment[Publication Type]) NOT (Letter[Publication Type]))
Embase	#7	P	'Chronic kidney failure'/exp OR ' Chronic renal failure ':ab,ti OR 'Chronic kidney disease':ab,ti
	#8	I	'Blood pressure monitoring'/exp OR ' Blood pressure regulation':ab,ti OR ' Blood pressure determination ':ab,ti OR ' Blood pressure control ':ab,ti OR ' Control blood p`ressure ':ab,ti
	#9	C	NA
	#10	O	'all cause mortality'/exp OR 'Cardiovascular diseases '/exp OR 'Chronic kidney disease '/exp OR ' Cardiovascular calcification':ab,ti OR 'cardiovascular disease assessment':ab,ti OR 'coronary artery atherosclerosis':ab,ti OR 'Cardiovascular disease':ab,ti OR 'Cardiovascular risk factor':ab,ti OR 'Cardiovascular calcification':ab,ti OR 'all-cause mortality':ab,ti OR ' Serum Creatinine':ab,ti OR ' GFR ':ab,ti OR 'eGFR':ab,ti OR ‘glomerular filtration rate’:ab,ti OR ‘Composite renal outcome’:ab,ti
	#11	S	'randomized controlled trial'/exp NOT review:it
	#12 = #7 AND #8 AND #10 AND #11	NA	('Chronic kidney failure'/exp OR ' Chronic renal failure ':ab,ti OR 'Chronic kidney disease':ab,ti) AND ('Blood pressure monitoring'/exp OR ' Blood pressure regulation':ab,ti OR ' Blood pressure determination ':ab,ti OR ' Blood pressure control ':ab,ti OR ' Control blood p`ressure ':ab,ti) AND ('all cause mortality'/exp OR 'Cardiovascular diseases '/exp OR 'Chronic kidney disease '/exp OR ' Cardiovascular calcification':ab,ti OR 'cardiovascular disease assessment':ab,ti OR 'coronary artery atherosclerosis':ab,ti OR 'Cardiovascular disease':ab,ti OR 'Cardiovascular risk factor':ab,ti OR 'Cardiovascular calcification':ab,ti OR 'all-cause mortality':ab,ti OR ' Serum Creatinine':ab,ti OR ' GFR ':ab,ti OR 'eGFR':ab,ti OR ‘glomerular filtration rate’:ab,ti OR ‘Composite renal outcome’:ab,ti) AND ('randomized controlled trial'/exp NOT review:it)

Abbreviation: NA, not applicable
