# Supplementary material for: Effects of intensive blood pressure control on mortality and cardiorenal function in chronic kidney disease patients
Source: Ren Fail. 2021 May 10;43(1):811–20. doi: 10.1080/0886022X.2021.1920427 (PMC8118417; doi:10.1080/0886022X.2021.1920427)
Supplement: Supplemental Material [file IRNF_A_1920427_SM3665.pdf]

Table S2. Inclusion/exclusion criteria of literature

PICOS	Inclusion	Exclusion
P	Participants were patients with CKD or included in a subgroup of CKD.	Children and pregnant women
I	Patients were assigned their trial intervention group, intensive versus standard BP control. Trial defnitions of intensive or standard BP targets were used.	Not available
C	No intervention, or with placebo or a lifestyle intervention.	Not available
O	One of the following outcomes must have been included: doubling of serum creatinine level or 50% reduction in GFR, composite renal outcome, cardiovascular outcomes, serious adverse events or all-cause mortality.	Not available
S	RCT irrespective of blinding or arm	1) Articles without peer reviewed or unpublished<br>2) Studies that were repeatedly published or had qualitative outcomes<br>3) Quasi-experimental studies and observational studies
