# Supplementary figures and images for: Effects of intensive blood pressure control on mortality and cardiorenal function in chronic kidney disease patients
Source: Ren Fail. 2021 May 10;43(1):811–20. doi: 10.1080/0886022X.2021.1920427 (PMC8118417; doi:10.1080/0886022X.2021.1920427)

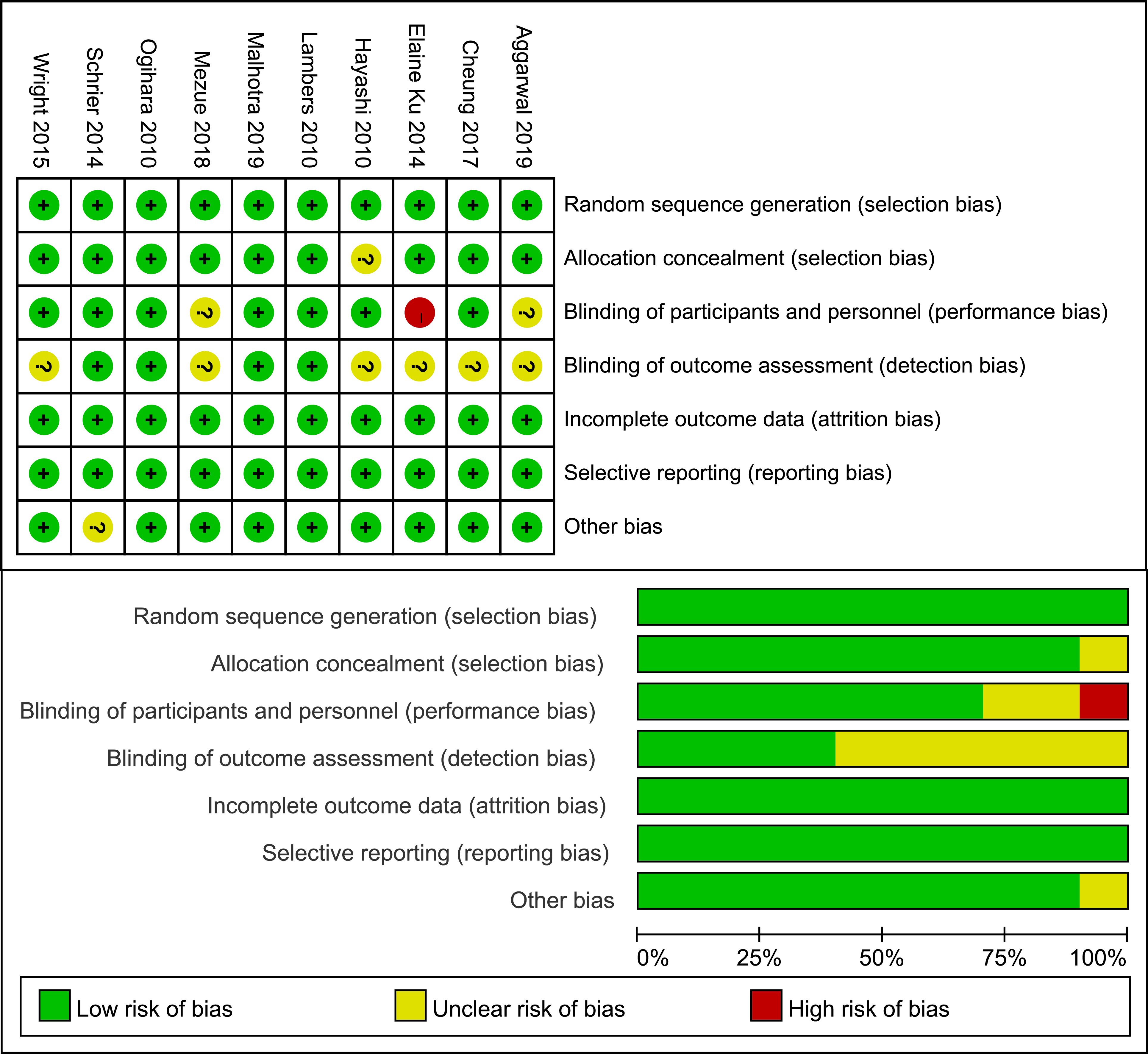

Supplement: Supplemental Material [file IRNF_A_1920427_SM3629.tif]

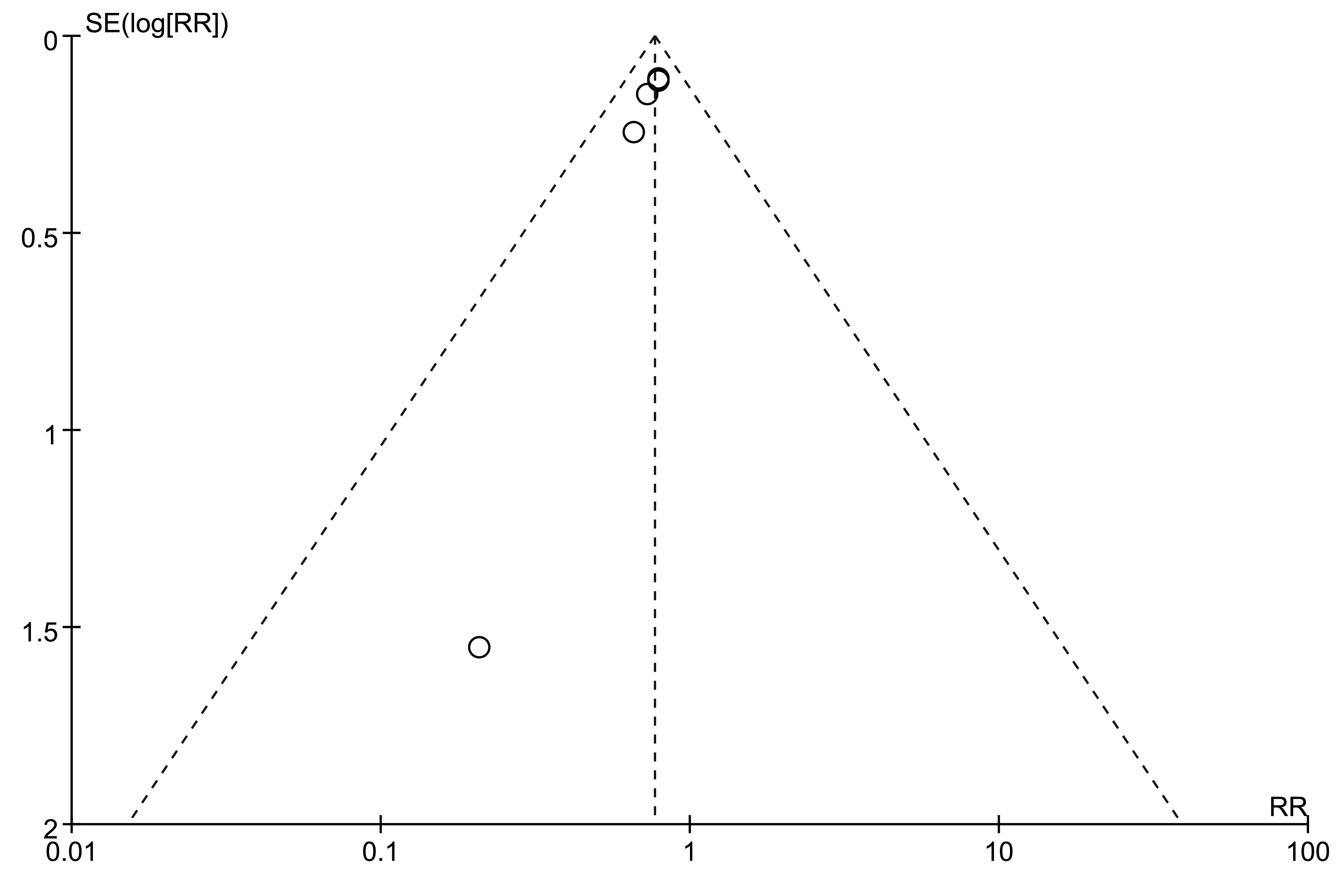

Supplement: Supplemental Material [file IRNF_A_1920427_SM3619.tif]

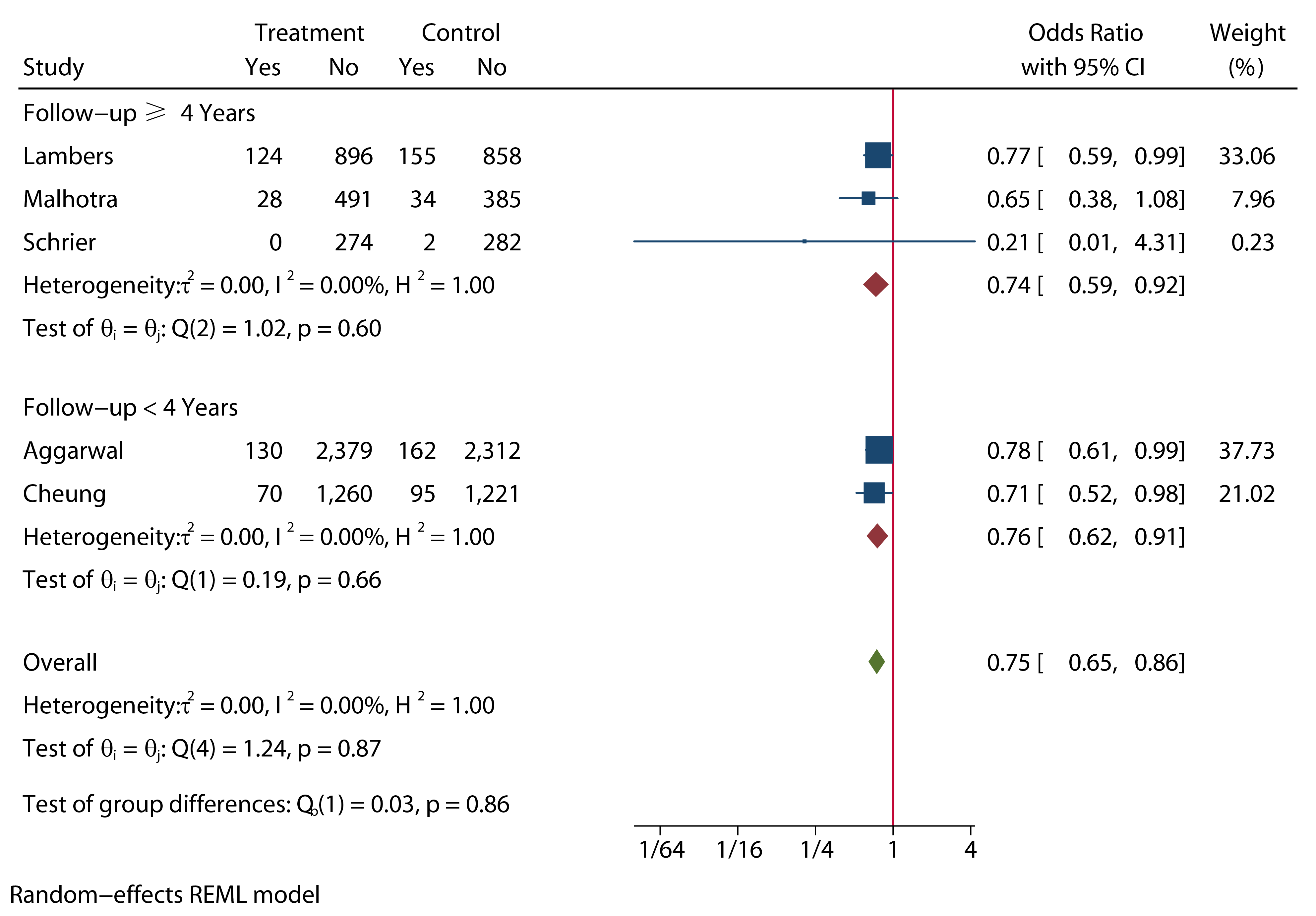

Supplement: Supplemental Material [file IRNF_A_1920427_SM3604.tif]

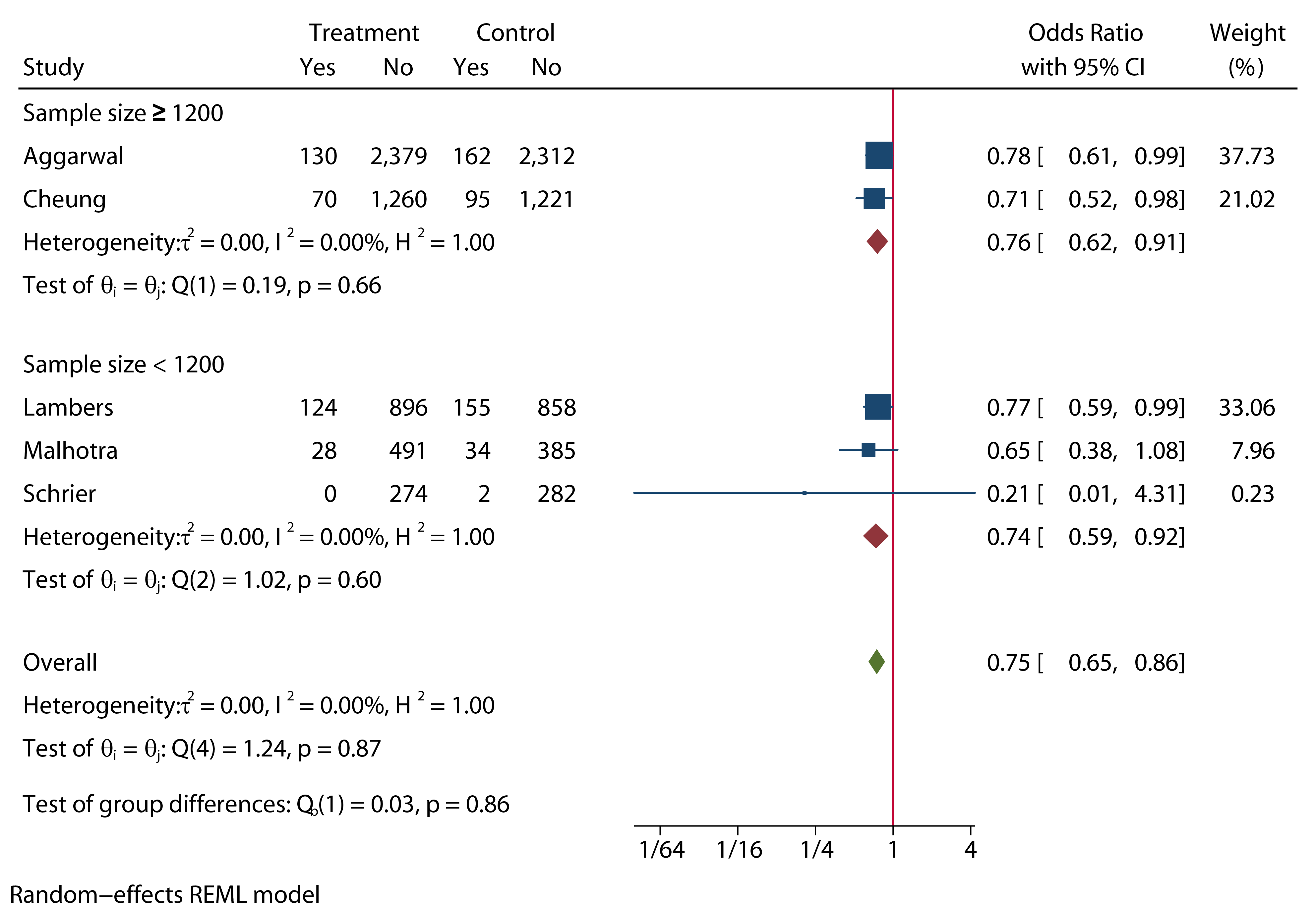

Supplement: Supplemental Material [file IRNF_A_1920427_SM3539.tif]

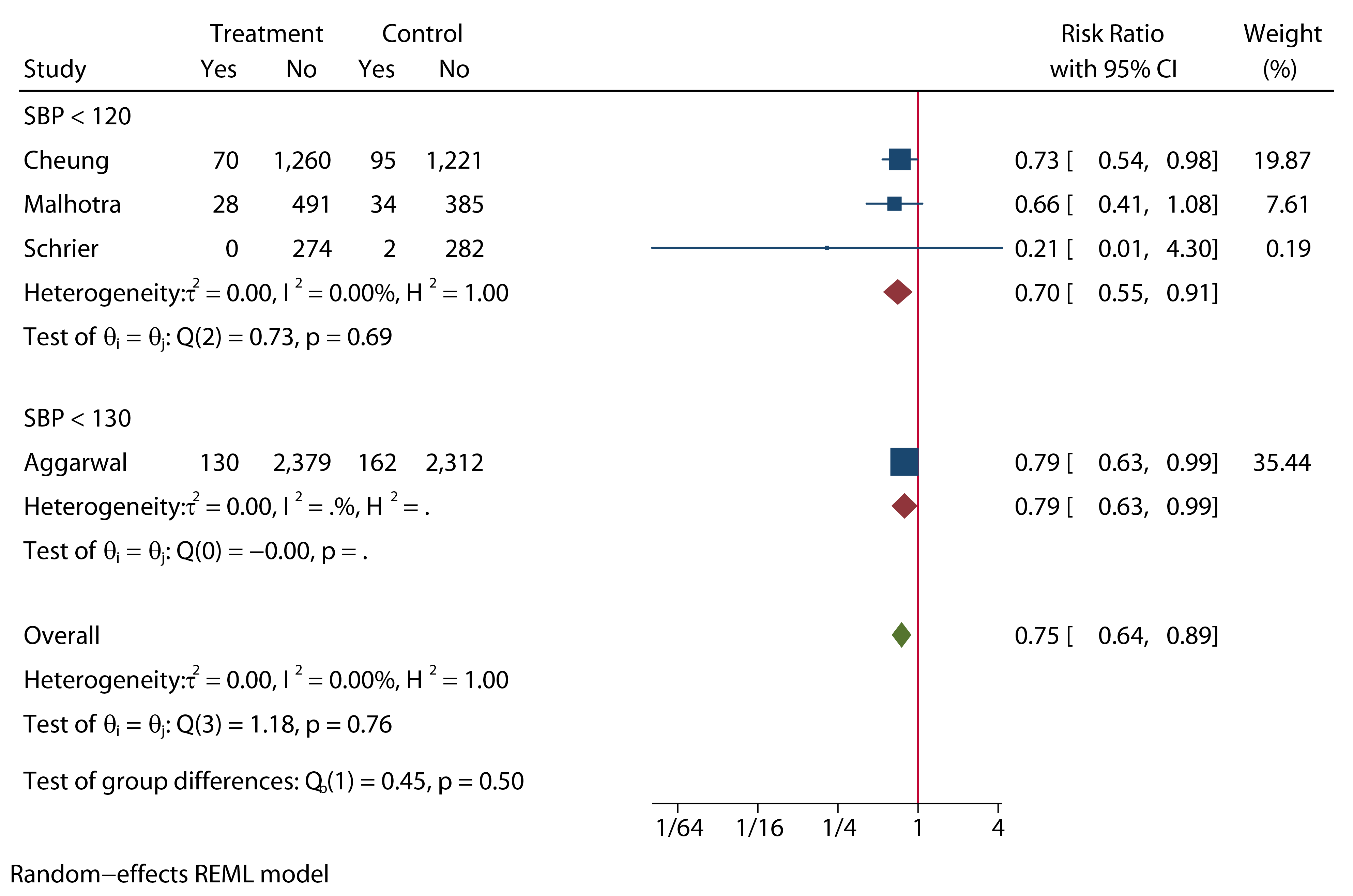

Supplement: Supplemental Material [file IRNF_A_1920427_SM3536.tif]

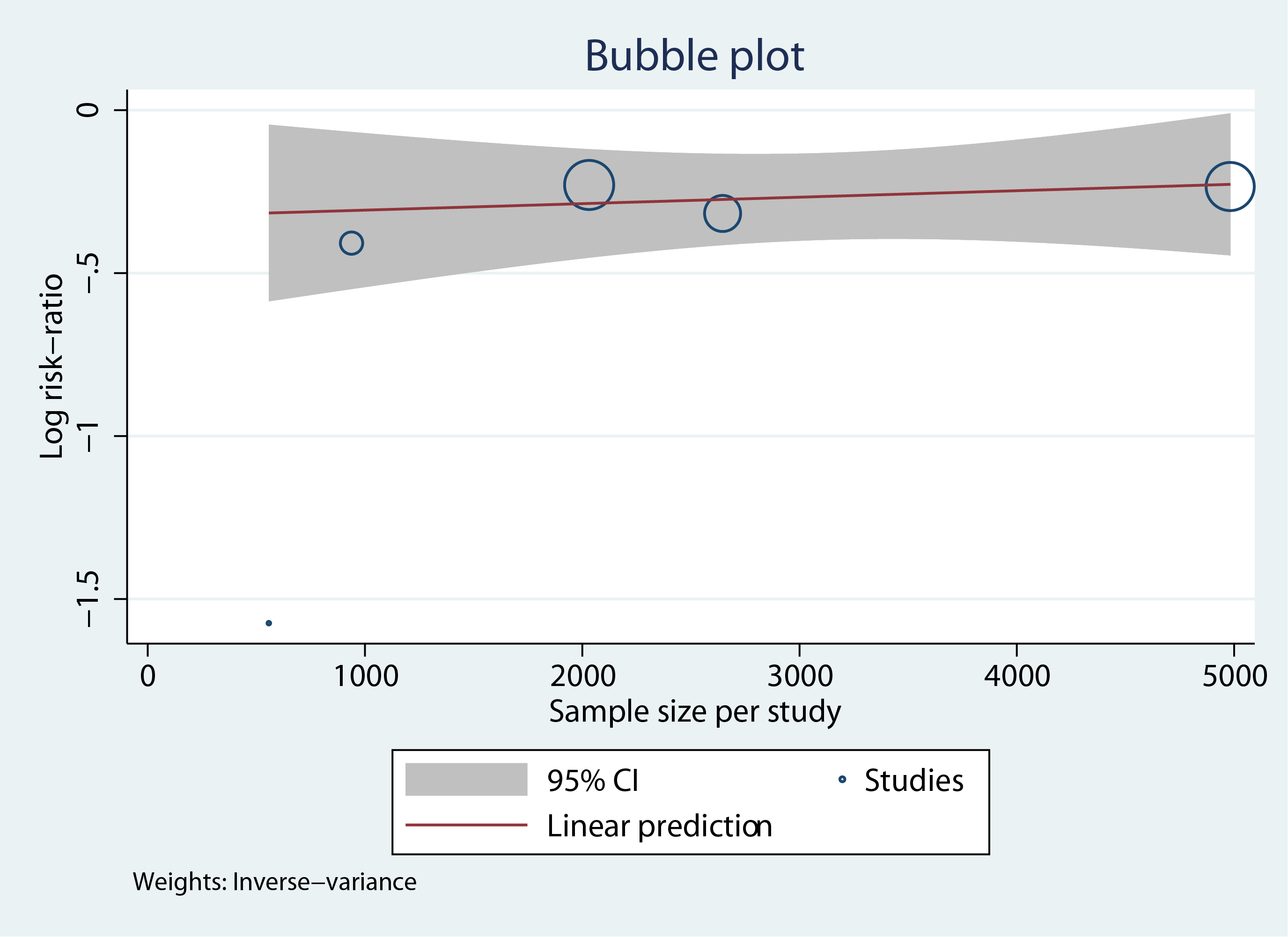

Supplement: Supplemental Material [file IRNF_A_1920427_SM3522.tif]

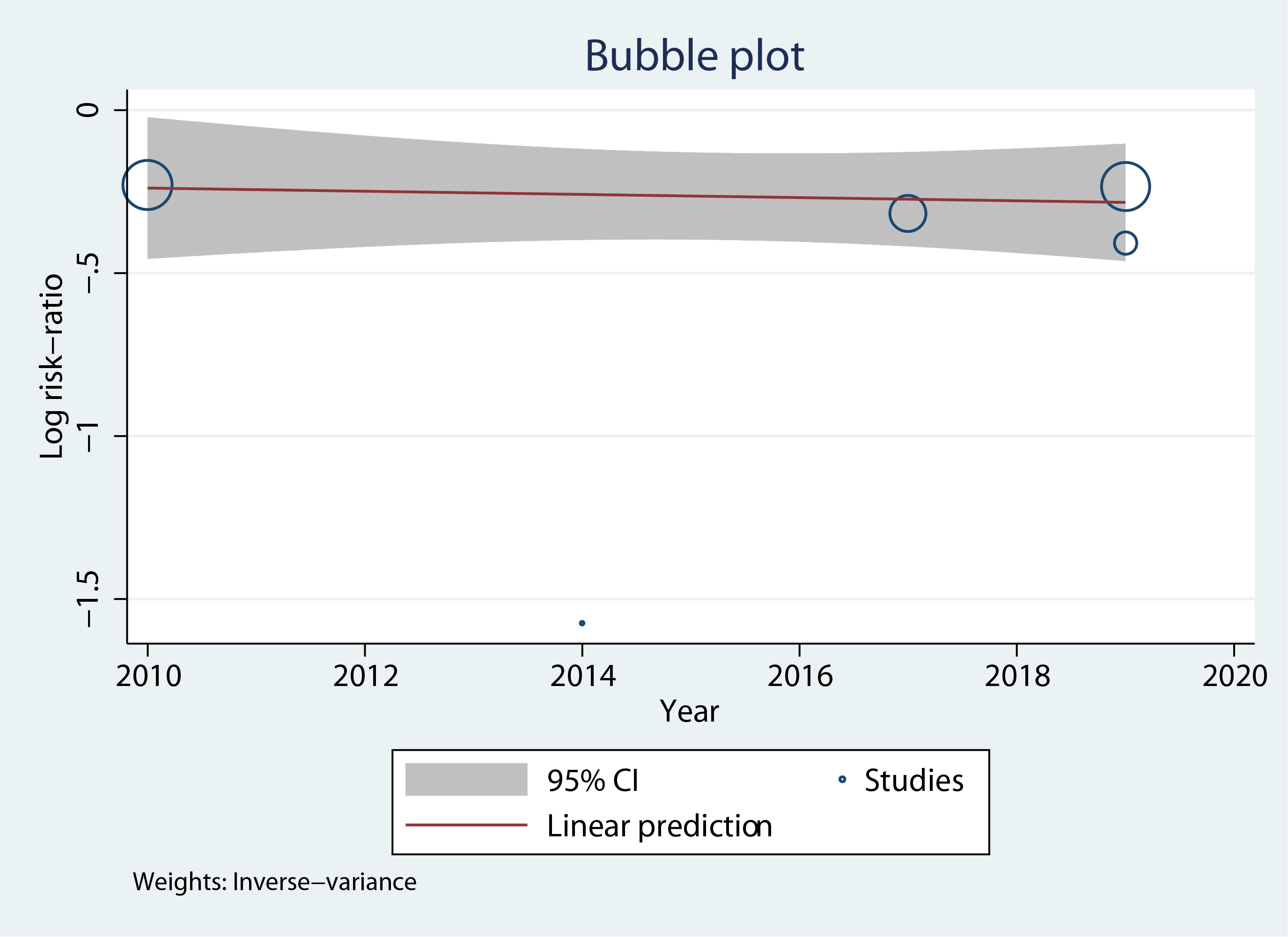

Supplement: Supplemental Material [file IRNF_A_1920427_SM3514.tif]

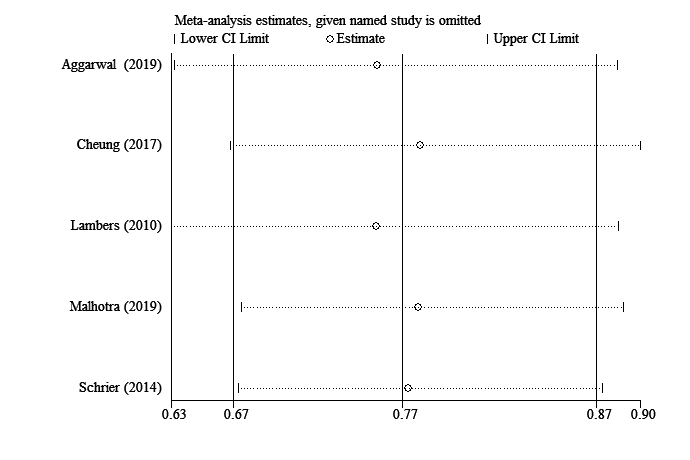

Supplement: Supplemental Material [file IRNF_A_1920427_SM3511.tif]

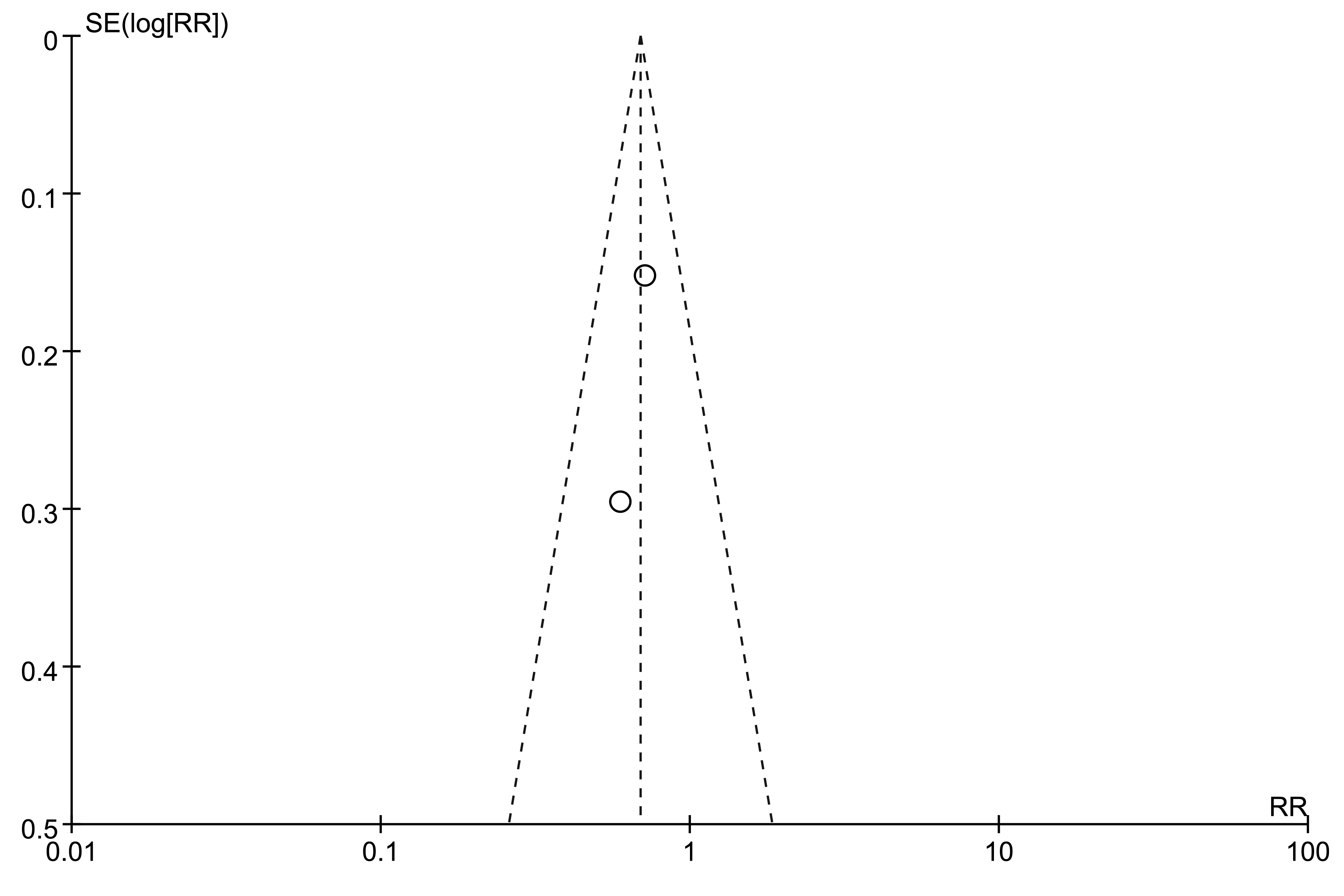

Supplement: Supplemental Material [file IRNF_A_1920427_SM3503.tif]

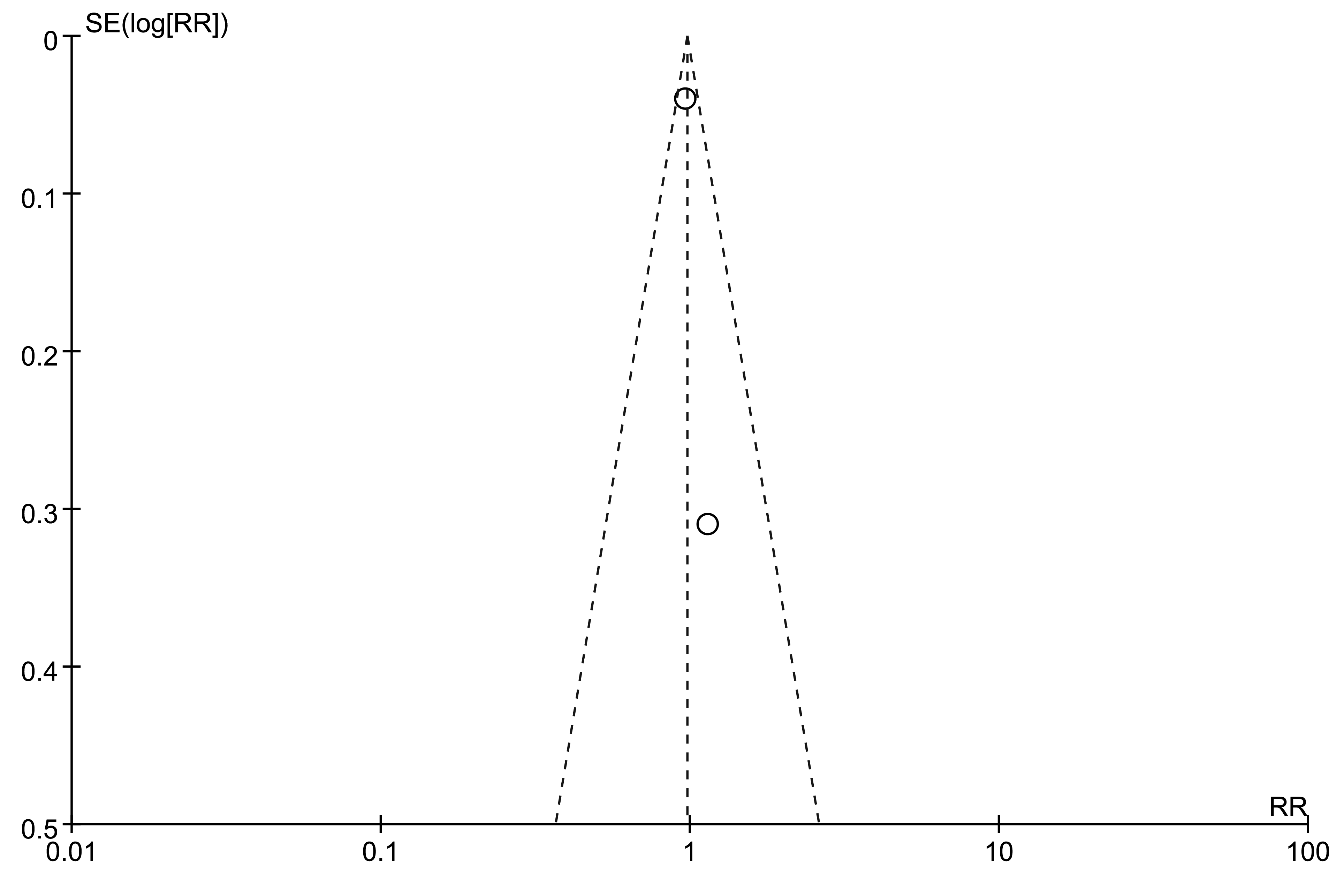

Supplement: Supplemental Material [file IRNF_A_1920427_SM3366.tif]

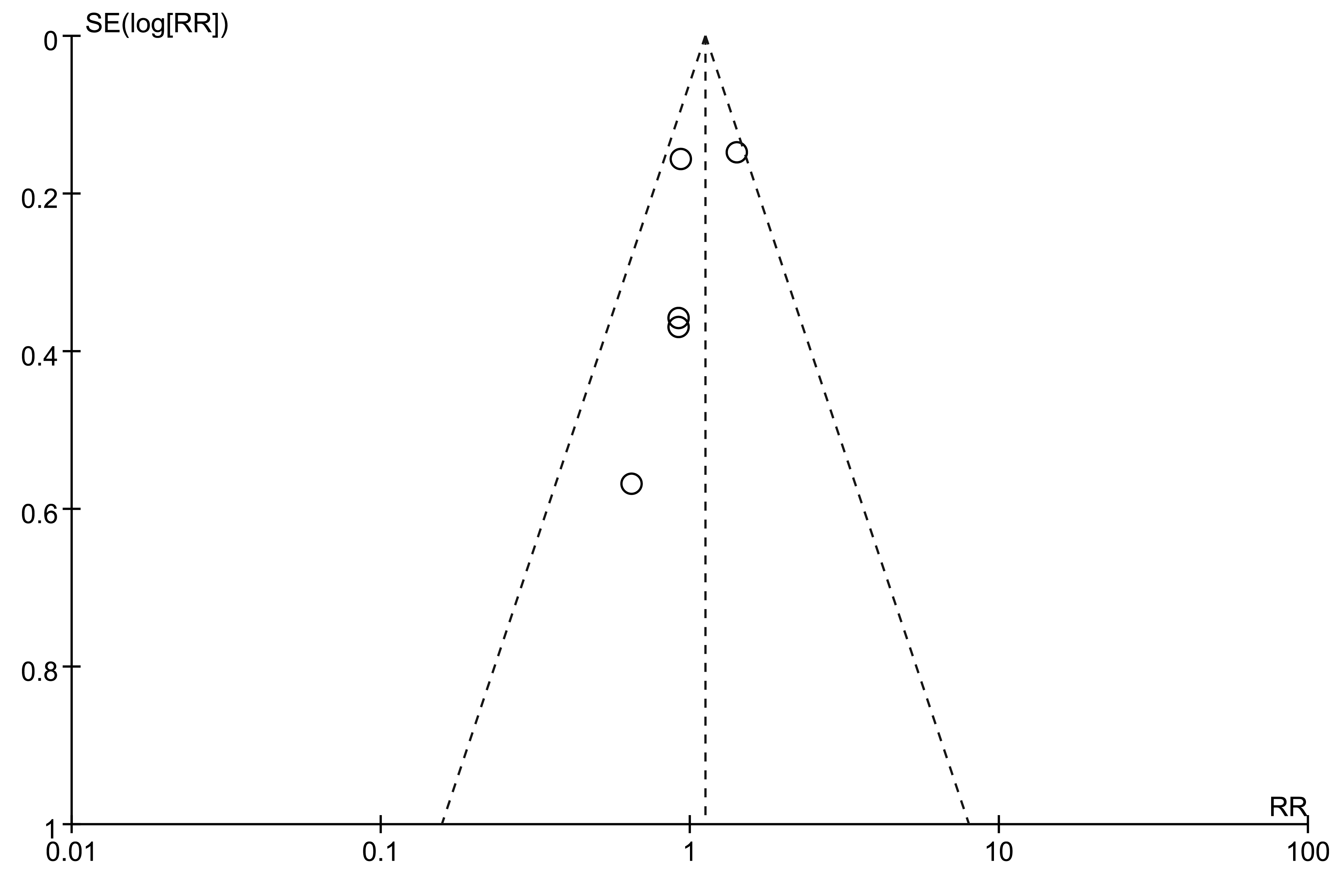

Supplement: Supplemental Material [file IRNF_A_1920427_SM3362.tif]

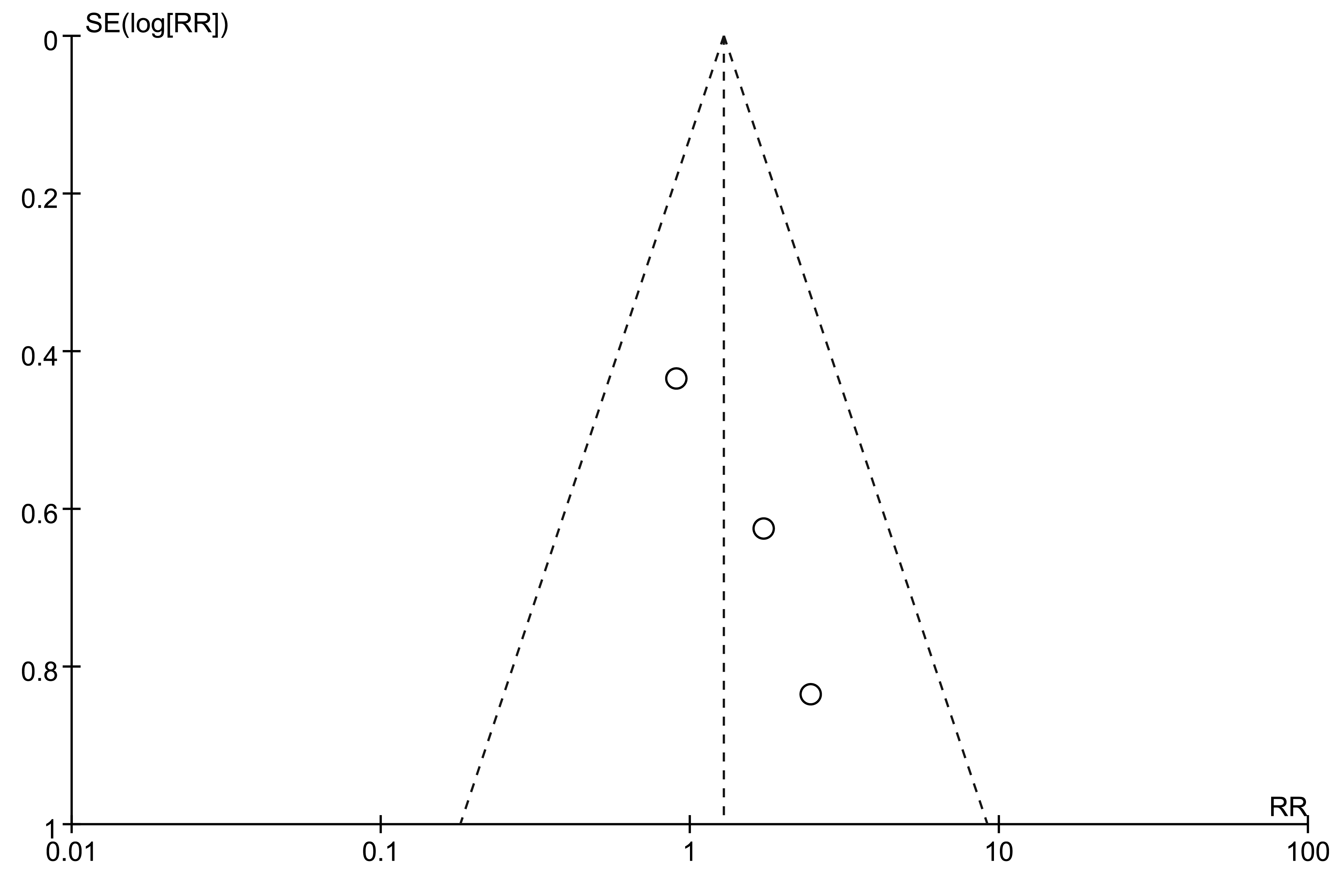

Supplement: Supplemental Material [file IRNF_A_1920427_SM3357.tif]

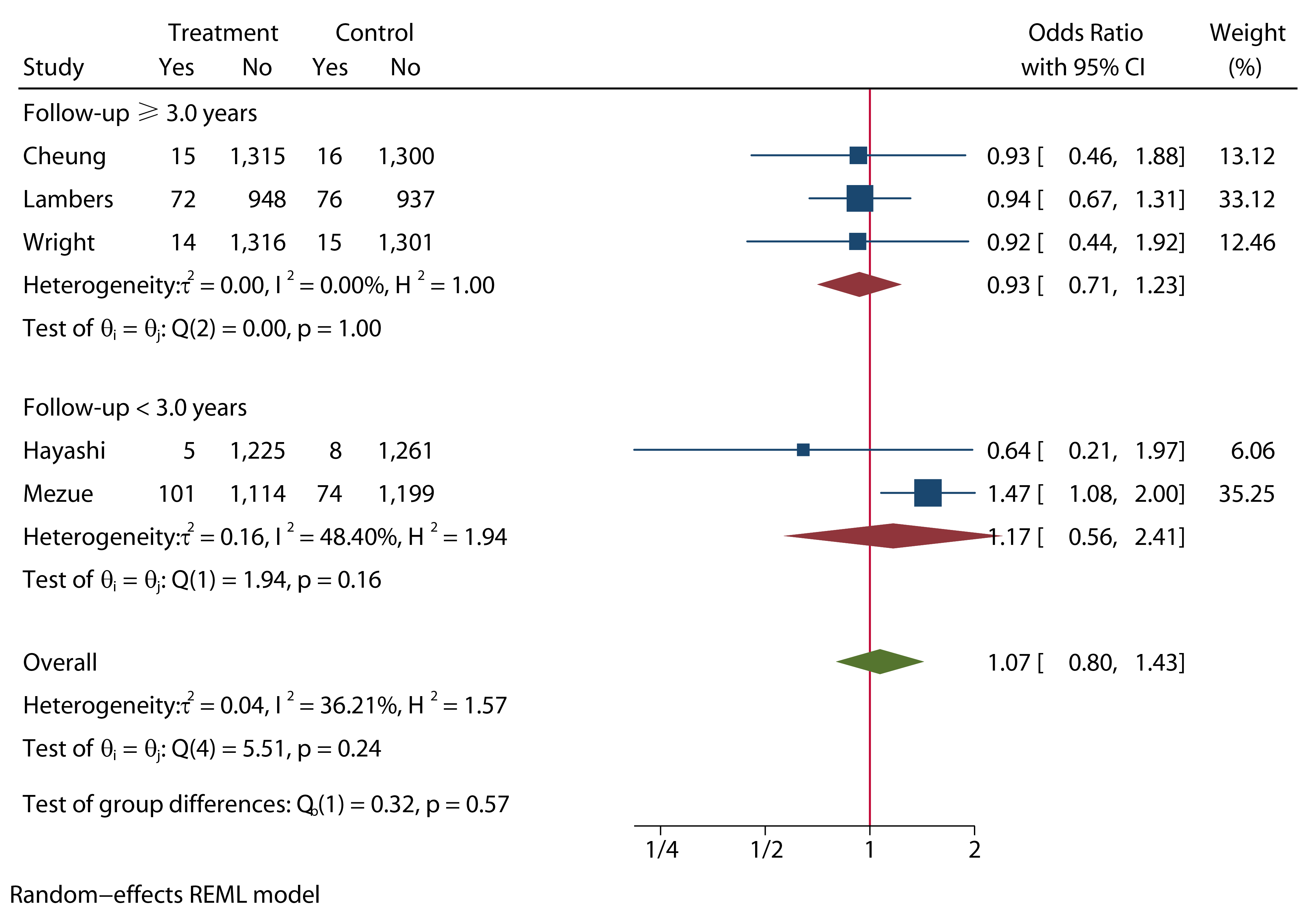

Supplement: Supplemental Material [file IRNF_A_1920427_SM3337.tif]

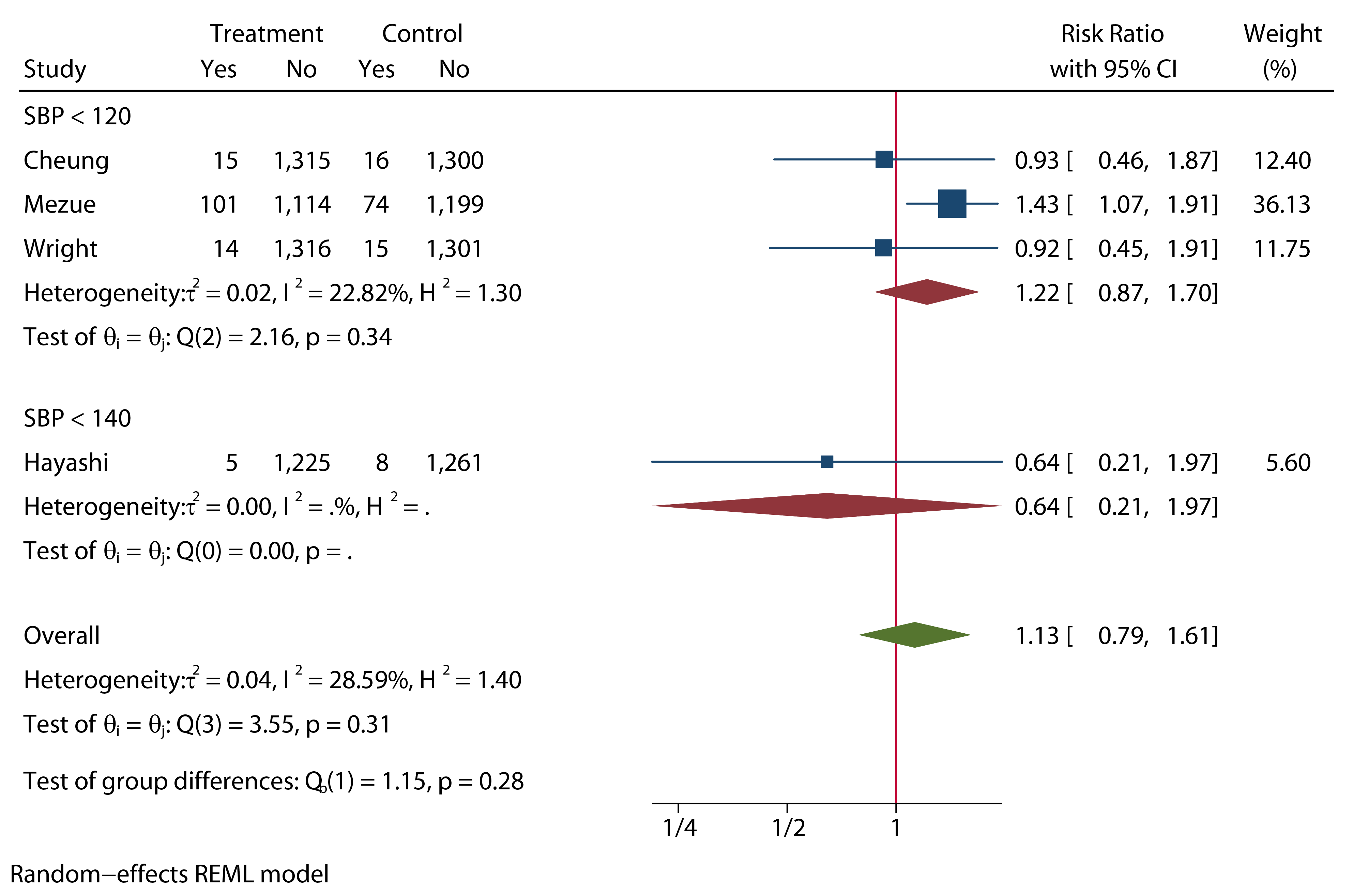

Supplement: Supplemental Material [file IRNF_A_1920427_SM3328.tif]

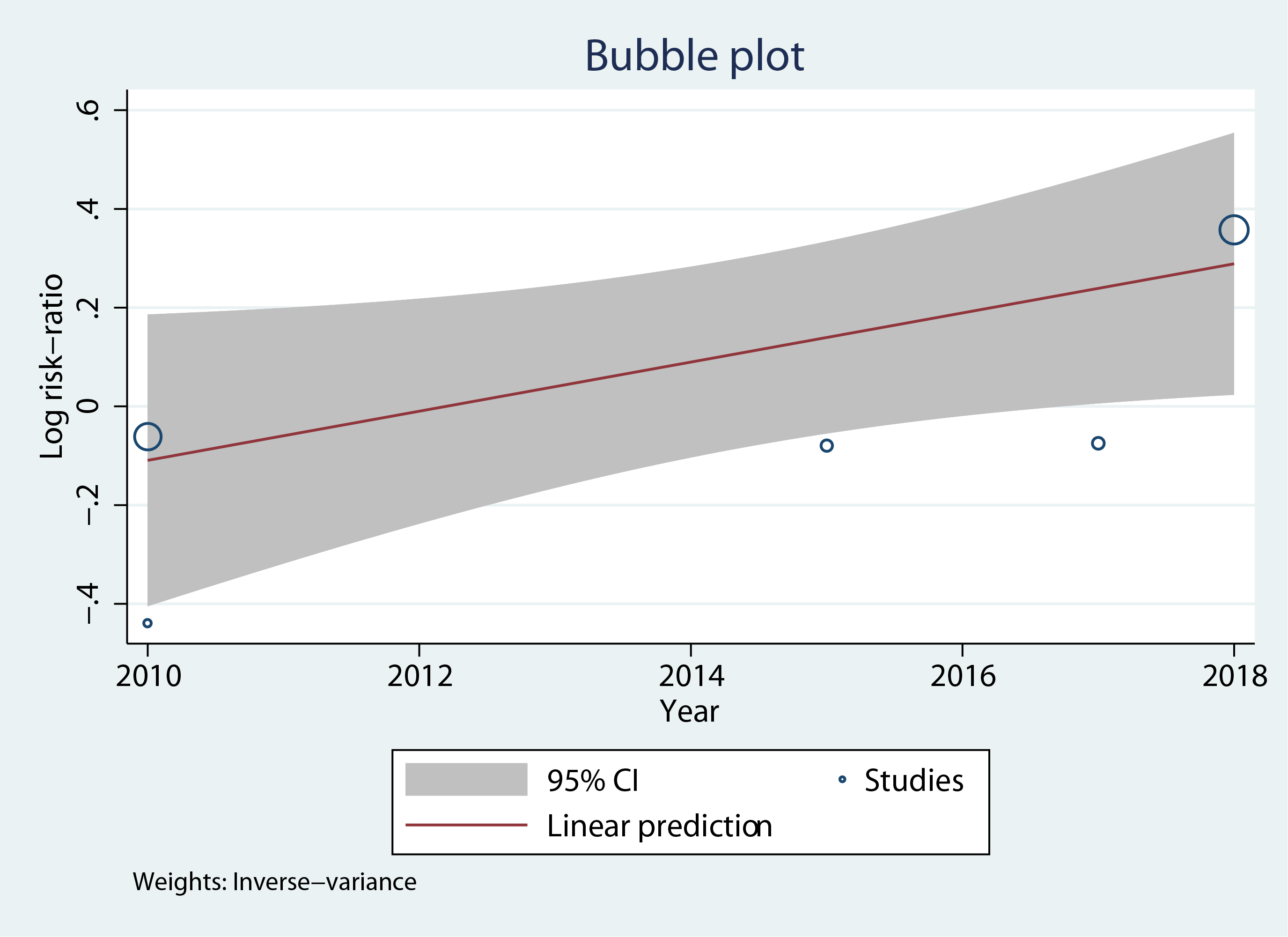

Supplement: Supplemental Material [file IRNF_A_1920427_SM3244.tif]

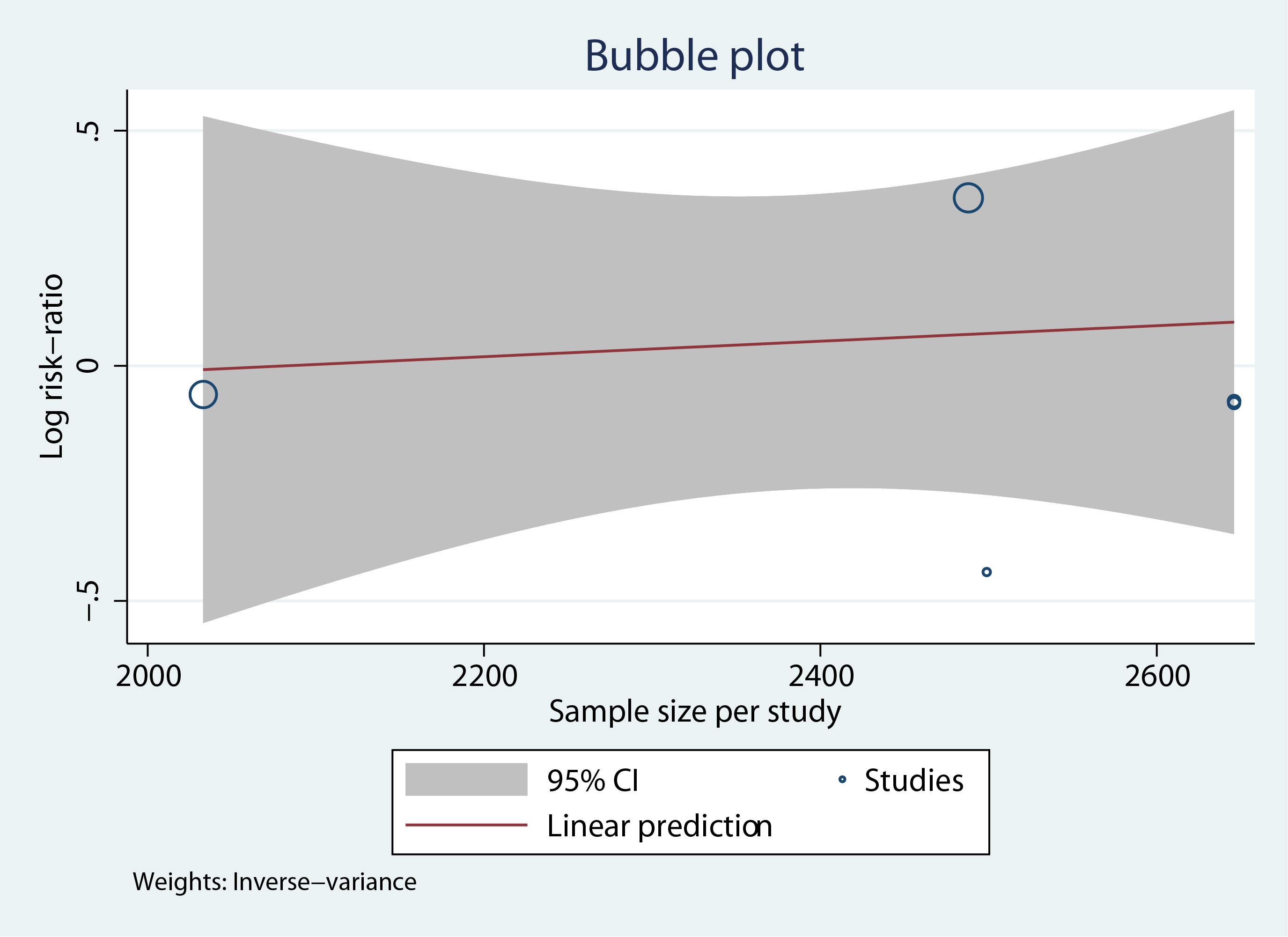

Supplement: Supplemental Material [file IRNF_A_1920427_SM3240.tif]

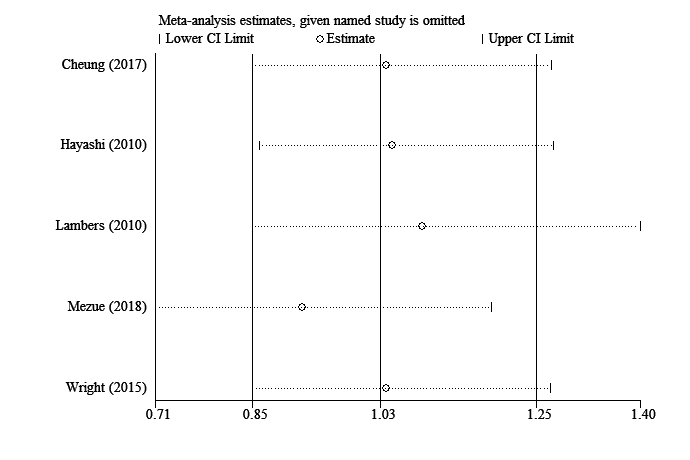

Supplement: Supplemental Material [file IRNF_A_1920427_SM3216.tif]

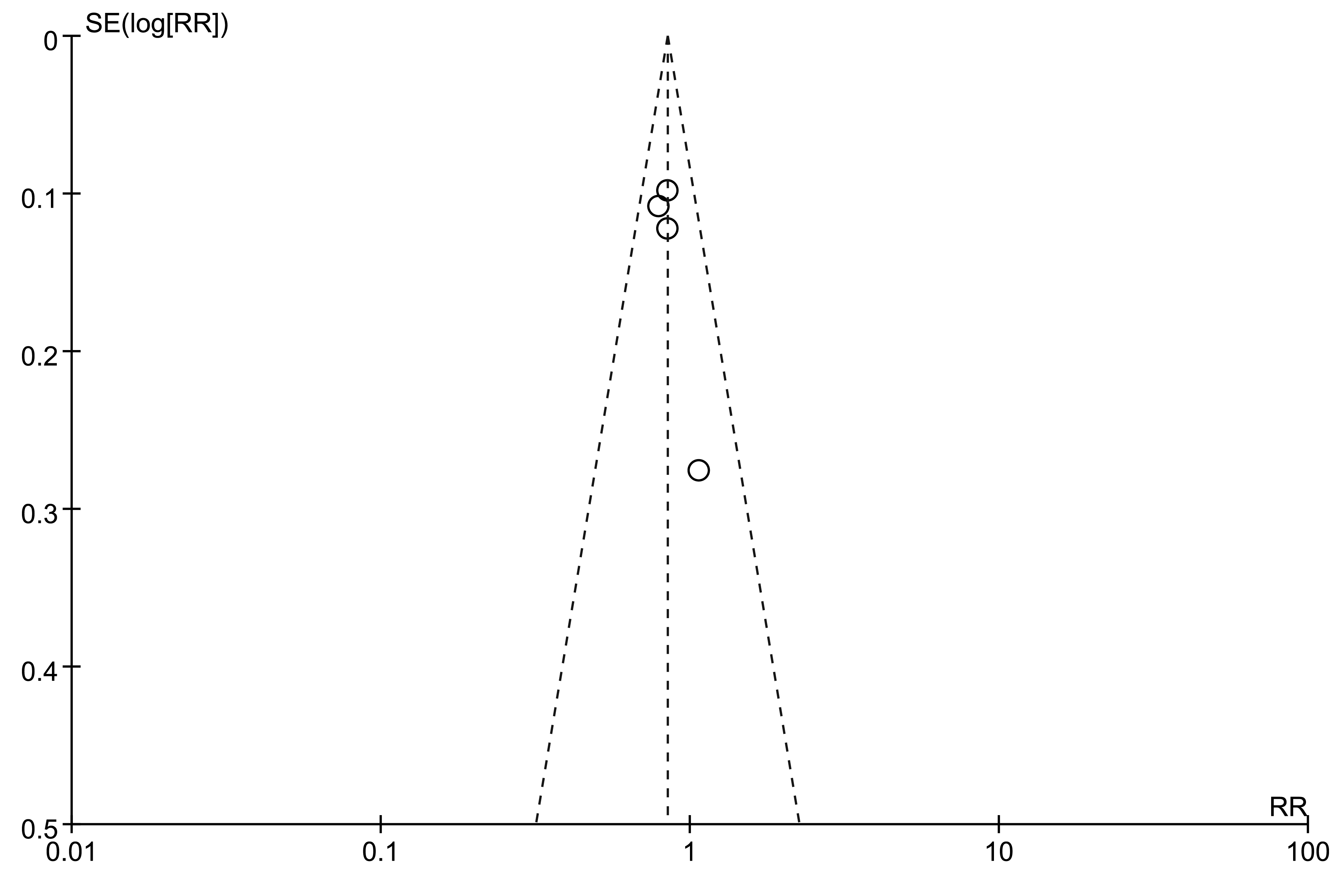

Supplement: Supplemental Material [file IRNF_A_1920427_SM3113.tif]
